# Body mass index and cancer risk among adults with and without cardiometabolic diseases: evidence from the EPIC and UK Biobank prospective cohort studies

**DOI:** 10.1186/s12916-023-03114-z

**Published:** 2023-11-23

**Authors:** Emma Fontvieille, Vivian Viallon, Martina Recalde, Reynalda Cordova, Anna Jansana, Laia Peruchet-Noray, Hannah Lennon, Alicia K. Heath, Dagfinn Aune, Sofia Christakoudi, Verena Katzke, Rudolf Kaaks, Elif Inan-Eroglu, Matthias B. Schulze, Lene Mellemkjær, Anne Tjønneland, Kim Overvad, Marta Farràs, Dafina Petrova, Pilar Amiano, María-Dolores Chirlaque, Conchi Moreno-Iribas, Sandar Tin Tin, Giovanna Masala, Sabina Sieri, Fulvio Ricceri, Salvatore Panico, Anne M. May, Evelyn M. Monninkhof, Elisabete Weiderpass, Marc J. Gunter, Pietro Ferrari, Heinz Freisling

**Affiliations:** 1 Nutrition and Metabolism Branch, International Agency for Research On Cancer (IARC-WHO), 25 Avenue Tony Garnier, CS 90627, 69366 Lyon, CEDEX 07 France; 2 Department of Clinical Sciences, Faculty of Medicine, University of Barcelona, Barcelona, Spain; 3 Department of Epidemiology and Biostatistics, School of Public Health, Imperial College London, London, UK; 4 Department of Nutrition, Oslo New University College, Oslo, Norway; 5 Department of Research, Cancer Registry of Norway, Oslo, Norway; 6 Department of Inflammation Biology, School of Immunology and Microbial Sciences, King’s College London, London, UK; 7 Department of Cancer Epidemiology, German Cancer Research Center (DKFZ), Heidelberg, Germany; 8 Department of Molecular Epidemiology, German Institute of Human Nutrition Potsdam-Rehbruecke, Nuthetal, Germany; 9 Institute of Nutritional Science, University of Potsdam, Nuthetal, Germany; 10 Danish Cancer Society Research Center, Copenhagen, Denmark; 11 Department of Public Health, University of Copenhagen, Copenhagen, Denmark; 12 Department of Public Health, Aarhus University, Aarhus, Denmark; 13 Unit of Nutrition and Cancer, Cancer Epidemiology Research Program, Institut Català d’Oncologia, Bellvitge Biomedical Research Institute (IDIBELL), 08908 L’Hospitalet de Llobregat, Spain; 14 Escuela Andaluza de Salud Pública (EASP), 18011 Granada, Spain; 15 Instituto de Investigación Biosanitaria Ibs.GRANADA, 18012 Granada, Spain; 16 Centro de Investigación Biomédica en Red de Epidemiología y Salud Pública (CIBERESP), 28029 Madrid, Spain; 17 Ministry of Health of the Basque Government, Sub Directorate for Public Health and Addictions of Gipuzkoa, 2013 San Sebastian, Spain; 18 Biodonostia Health Research Institute, Epidemiology of Chronic and Communicable Diseases Group, 20014 San Sebastián, Spain; 19 Spanish Consortium for Research On Epidemiology and Public Health (CIBERESP), Instituto de Salud Carlos III, Madrid, Spain; 20 Department of Epidemiology, Regional Health Council, IMIB-Arrixaca, Murcia University, Murcia, Spain; 21 Navarra Public Health Institute, Pamplona, Spain; 22 Navarra Institute for Health Research (IdiSNA), Pamplona, Spain; 23 Cancer Epidemiology Unit, Oxford Population Health, University of Oxford, Oxford, UK; 24 Institute for Cancer Research, Prevention and Clinical Network (ISPRO), Florence, Italy; 25 Epidemiology and Prevention Unit, Fondazione IRCCS Istituto Nazionale Dei Tumori Di Milano, Milan, Italy; 26 Department of Clinical and Biological Sciences, Centre for Biostatistics, Epidemiology, and Public Health, University of Turin, Turin, Italy; 27 Department of Clinical Medicine and Surgery, Federico II University, Naples, Italy; 28 Julius Center for Health Sciences and Primary Care, University Medical Center Utrecht, Utrecht University, Utrecht, The Netherlands

**Keywords:** Obesity, Type 2 diabetes, Cardiovascular diseases, Comorbidities, Obesity-related cancers, Multimorbidity

## Abstract

**Background:**

Whether cancer risk associated with a higher body mass index (BMI), a surrogate measure of adiposity, differs among adults with and without cardiovascular diseases (CVD) and/or type 2 diabetes (T2D) is unclear. The primary aim of this study was to evaluate separate and joint associations of BMI and CVD/T2D with the risk of cancer.

**Methods:**

This is an individual participant data meta-analysis of two prospective cohort studies, the UK Biobank (UKB) and the European Prospective Investigation into Cancer and nutrition (EPIC), with a total of 577,343 adults, free of cancer, T2D, and CVD at recruitment. We used Cox proportional hazard regressions to estimate multivariable-adjusted hazard ratios (HRs) and 95% confidence intervals (CIs) for associations between BMI and incidence of obesity-related cancer and in turn overall cancer with a multiplicative interaction between BMI and the two cardiometabolic diseases (CMD). HRs and 95% CIs for separate and joint associations for categories of overweight/obesity and CMD status were estimated, and additive interaction was quantified through relative excess risk due to interaction (RERI).

**Results:**

In the meta-analysis of both cohorts, BMI (per ~ 5 kg/m^2^) was positively associated with the risk of obesity-related cancer among participants without a CMD (HR: 1.11, 95%CI: 1.07,1.16), among participants with T2D (HR: 1.11, 95% CI: 1.05,1.18), among participants with CVD (HR: 1.17, 95% CI: 1.11,1.24), and suggestively positive among those with both T2D and CVD (HR: 1.09, 95% CI: 0.94,1.25). An additive interaction between obesity (BMI ≥ 30 kg/m^2^) and CVD with the risk of overall cancer translated into a meta-analytical RERI of 0.28 (95% CI: 0.09–0.47).

**Conclusions:**

Irrespective of CMD status, higher BMI increased the risk of obesity-related cancer among European adults. The additive interaction between obesity and CVD suggests that obesity prevention would translate into a greater cancer risk reduction among population groups with CVD than among the general population.

**Supplementary Information:**

The online version contains supplementary material available at 10.1186/s12916-023-03114-z.

## Background 

The prevalence of overweight and obesity (body mass index, BMI ≥ 25 kg/m^2^) has increased globally in recent decades, from 20% in 1975 to over 39% in 2016 [1]. Overweight and obesity increase the risk of non-communicable diseases (NCDs) including cardiometabolic diseases (CMD) [2]—such as type 2 diabetes (T2D) and cardiovascular diseases (CVD)—and cancer [3]. A high BMI, a surrogate measure of body fatness, is an established risk factor for at least 13 types of cancer, including adenocarcinoma of the breast (postmenopausal women), colorectal, gallbladder, kidney, liver, meningioma, multiple myeloma, oesophageal, ovarian, pancreatic, thyroid, gastric cardia (upper stomach), and corpus uteri [1, 3]. These cancers alone (described hereafter as obesity-related cancers) comprise approximately 37% of the total global burden of cancer (based on GLOBOCAN 2020 data [4]) and at present impact more on populations in high-income countries, where 67% of all obesity-related cancers are diagnosed [5]. The co-occurrence of CMD and cancer in an individual has become common, resulting in multimorbidity [6]. In turn, T2D has also been associated with increased risk of cancers of the colorectum, pancreas, liver, gallbladder, breast, and corpus uteri [7–12], all of which are also obesity-related cancers. Emerging evidence suggests that CVD might be an independent risk factor for certain cancers even after accounting for shared risk factors including BMI [13–15]. T2D and CVD also share common biological pathways with cancer, such as inflammation, oxidative stress, or hormonal processes [6]. Taken together, the presence of one or more of these CMDs may exacerbate the negative health effects of overweight and obesity in the development of cancer, though this is not well understood.

Prior studies focused on the BMI–cancer association in the general population, but little attention has been given to the co-occurrence of CMD [16–18]. In a 2021 meta-analysis of six cohort studies in patients with T2D, a higher BMI was associated with an increased risk of total cancer and breast cancer, a suggestive increased risk of colorectal cancer, but not with the risk of cancers of the prostate and pancreas [19]. Similar studies investigating BMI–cancer associations among population groups with CVD are largely lacking. Furthermore, joint associations of overweight and/or obesity and CMD with cancer risk have not been studied with the exception of our companion study by Recalde et al. [16]. Considering that prevalent cases of both CVD and T2D are likely to increase substantially as a result of population growth and aging [20, 21], it is important to investigate potential differences in the BMI–cancer relationship among population groups affected by CMD [22]. Such knowledge would enable evaluating the burden of cancer in populations with different prevalence of overweight and obesity, T2D, and CVD and inform health recommendations such as cancer screening programs and lifestyle interventions to prevent and control the co-occurrence of multiple NCDs in a targeted population.

We evaluated whether the association between BMI and obesity-related cancer risk differs among adults with and without CMD in the European Prospective Investigation into Cancer and Nutrition (EPIC) and the UK Biobank (UKB) prospective cohort studies. We investigated potential interaction between BMI and CMD and risk of obesity-related cancer on both the multiplicative and additive scale. In this context, we also investigated total cancer (all cancers combined) as a secondary outcome.

## Methods

### Study population

The UKB is a prospective cohort study of around 500,000 individuals aged 40–69 years enrolled between 2006 and 2010 across 22 centers located in England, Scotland, and Wales. At recruitment, information on socio-demographic characteristics, lifestyle factors, diet, anthropometry, and biological samples were collected [23]. Participants were followed from recruitment until the earliest of cancer, death, loss to follow-up, or end of the study period (between February 2020 and March 2021 depending on center).

EPIC is a prospective cohort with approximately 521,000 adults mostly aged 35–69 years at enrolment (between 1992 and 2000) from 23 research centers across 10 European countries (Denmark, France, Germany, Greece, Italy, Norway, Spain, Sweden, the Netherlands, and the UK) [24]. At recruitment, participants completed questionnaires covering socio-demographic, lifestyle, diet, and reproductive factors and anthropometric measurements and blood samples were also collected [24]. Participants were followed from recruitment until end of follow-up (i.e., last date of center- and event-specific ascertainment of CVD, T2D, or cancer, whichever came first), death, loss to follow-up, or end of the study [25].

As shown in Additional File 1: Figures S1 and S2, we excluded participants who had cancer, CVD, or T2D prior to enrolment in both UKB and EPIC. The rationale to exclude participants with a history of CVD or T2D was to avoid potential reverse causation (i.e., CVD or T2D affecting BMI at recruitment). We further excluded participants with missing values in any covariate (~ 21% and 3% in UKB and EPIC, respectively). All analyses were performed in a sample restricted to participants with no missing data (complete-case analysis). In EPIC, participants from France, Greece, and Norway were excluded due to the lack of data on incident events of CVD or T2D. Last, we excluded participants from Sweden due to uncertain dates for a majority of T2D diagnoses.

### Anthropometry

Weight and height were measured by trained staff in both cohorts using comparable procedures [24]. In UKB, height was measured to the nearest centimeter using a Seca 202 stadiometer, and body weight to the nearest 0.1kg using a Tanita BC-418 body composition analyser [23]. In EPIC, body measurements were obtained using a standard protocol in all centres, except in Oxford (UK) where measurements were self-reported [26]. Depending on study center, height was measured to the nearest 0.1, 0.5, or 1.0 cm and weight to the nearest 0.1 kg [27]. BMI was calculated as weight/height^2^ (kg/m^2^) and categorized according to WHO definitions [1] into overweight/obesity (BMI ≥ 25 kg/m^2^) and obesity (BMI ≥ 30 kg/m^2^).

### Ascertainment of cardiometabolic diseases

Incident cases of both CVD and T2D were coded using the 10th Edition of the International Classification of Diseases (ICD-10). In both cohorts, CVD was defined as a composite of ischemic heart diseases (I20-I25), atrial fibrillation (I48), and cerebrovascular disease (I60-I69), and T2D was defined as E11 (Additional File 1: Table S1) [28].

In the UKB, cases for both CVD and T2D were identified via linked hospital admissions records (primary diagnosis). The inpatient hospital data were obtained through linked medical records, mapped across England, Scotland, and Wales using the Hospital Episode Statistics in England, Scottish Morbidity Record, and Patient Episode Database for Wales.

In EPIC, the diagnoses of CVD were ascertained within the framework of the EPIC-Heart study using active follow-up through questionnaires, medical records, hospital morbidity registers, contact with medical professionals, retrieving and assessing death certificates, or verbal autopsy [29]. T2D cases were identified within the framework of the EPIC-Interact through a review of several sources including self-report, linkage to primary care registers, secondary care registers, medication use, hospital admissions, and mortality data, depending on the center [28]. Both EPIC-Heart and EPIC-InterAct were designed as case-cohort studies nested in the full EPIC cohort; however, the nested case-cohort design of these studies was not used in the current analysis.

### Ascertainment of cancer

The outcomes of interest were the occurrence of any first primary malignant cancer (excluding non-melanoma skin cancer and in situ cancers), combined as overall cancer, and obesity-related cancers. Obesity-related cancers were defined as meningioma, multiple myeloma, adenocarcinoma of the esophagus, and cancers of the thyroid, postmenopausal breast, gallbladder, stomach, liver, pancreas, kidney, ovary, uterus, colon, and rectum (colorectal) [3]. Type of incident cancer cases were coded according to ICD-10 and information on tumor morphology and histology was ascertained using the 3rd Edition of the International Classification of Diseases for Oncology (ICD-O-3), detailed in Additional File 1: Table S1. In UKB, data on cancer diagnoses were provided by NHS Digital and Public Health England for participants from England and Wales and by NHS Central Register (NHSCR) for participants residing in Scotland and were ascertained through cancer registries. In EPIC, cases were identified by linkage to cancer registries for Denmark, Italy, the Netherlands, Spain, and the UK, and through a combination of health insurance records, cancer pathology registries, and active follow-up in Germany.

### Statistical analysis

We used Cox proportional hazards regression to estimate cause-specific hazard ratios (HRs) and 95% confidence intervals (CIs) for associations with obesity-related cancers and all-cancers per 1 standard deviation (SD) increment in BMI (~ 5 kg/m^2^). The entry time was age at recruitment and the exit time was age at first primary cancer diagnosis, end of follow-up, loss to follow-up, or death, whichever occurred first. Deaths from any cause were modelled as a censored observation. Follow-up for CVD and T2D, i.e., the CMD, also started at age at recruitment and the exit time was the same as described for cancer.

Our base model was stratified by center of recruitment, age (5-year categories), and sex and adjusted for educational level, alcohol consumption, smoking status, height, physical activity, diet score, and in women additionally for use of hormone therapy and menopausal status (Additional File 1: Table S2)*.* The menopausal status variable was allowed to change from pre- to post-menopausal among women, who turned 55 years during follow-up [30].We used a directed acyclic graph (DAG) [31] to identify confounding variables (Additional File 1: Figure S3). Our “overall adjusted model” further included CVD and T2D and their duration (time-varying accounting for non-linearity through natural splines) (Additional File 1: Figure S4). CVD and T2D were modelled as a time-varying variable with four categories (1: neither CVD nor T2D; 2: T2D; 3: CVD; and 4: T2D and CVD). Our main model further included a multiplicative interaction term between BMI (continuous) and the CMD variable (time-varying categorical) and estimates for the four categories were reported. We performed a likelihood ratio test to evaluate the multiplicative interaction between BMI and the CMD variable by comparing a model with the interaction term to a nested model without the interaction term.

We next evaluated the separate and joint associations of overweight and/or obesity (BMI ≥ 25 kg/m^2^ or BMI ≥ 30 kg/m^2^) and CMD (T2D, CVD, or both) with the risk of obesity-related cancer and total cancer. We created three variables each with four exclusive categories of combinations of overweight (and alternatively obesity) and in turn T2D, CVD, and both CMDs: (1) BMI < 25 kg/m^2^, without CMD of interest (reference); (2) BMI < 25 kg/m^2^, with CMD of interest; (3) BMI ≥ 25 kg/m^2^, without CMD of interest; and (4) BMI ≥ 25 kg/m^2^, with CMD of interest (joint effect). Based on this categorization, we quantified additive interaction for each of the three variables with the relative excess risk due to interaction (RERI) (as recommended in the STROBE statement [32, 33]) of the joint effect. The RERI was estimated as RERI_RR_ = RR_11_ – RR_10_ – RR_01_ + 1, with RR_11_ the relative risk of being exposed to both factors (e.g., overweight/obesity and T2D), RR_10_ exposed to one of the factors (overweight/obesity) and RR_01_ to the other one (e.g., T2D). Estimations of 95% CI were based on the delta method described by Hosmer and Lemeshow [34]. A RERI of 0 means a lack of additive interaction. The model was adjusted for the same variables as our base model and further adjusted for the two binary variables of the other two CMD statuses not studied [35].

All models were fitted in each of the two cohorts separately and results then combined using random-effects meta-analysis [36]. The proportion of total variability due to between-study heterogeneity was assessed by *I*^2^.

All analyses were also performed stratified by sex. To investigate potential biases, the following sensitivity analyses were carried out. First, to investigate uncontrolled confounding and/or collider bias due to conditioning on CMD, we considered non-obesity-related cancers as a negative-control outcome, for which a null association of obesity with non-obesity-related cancer was expected. Second, we additionally adjusted our main models for CMD treatment (use of metformin and/or statins) to assess its impact on risk estimates. The data on CMD treatment were available only in the UKB. Third, to assess residual confounding by smoking, we ran the main models among never smokers only. Lastly, we also evaluated potential collinearity between BMI and height by replacing height with residuals of height, which we computed by regressing BMI on height. Comparing our main models to a model with adjustment for residuals of height instead of height by likelihood ratio test indicated that both models were equivalent.

Statistical tests were two-sided, and *p*-values < 0.05 were considered statistically significant. All analyses were performed using R version 4.1.2. *Epi* and *InteractionR* packages were used for the main analyses.

## Results

Of a total of 577,343 study participants (UKB *n* = 344,094; EPIC *n* = 233,249), 53% and 62% were women in the UKB and EPIC cohorts, respectively. Baseline characteristics of the two study populations are reported, respectively, in Tables 1 and 2. In both cohorts, study participants with obesity (BMI ≥ 30 kg/m^2^) tended to have a lower educational level and were less physically active as compared to the other study participants. In UKB, during a median follow-up of 10.9 years (interquartile range, IQR: 10.1 – 11.7), a total of 32,549 first primary cancers (9.5% of participants) occurred, including 12,526 obesity-related cancers (of which 66% were in women). In EPIC, during a median follow-up of 10.9 years (IQR: 9.4 – 12.2), 19,833 first primary cancers (8.3% of participants) occurred, including 7892 obesity-related cancers (of which 76% were in women).

**Table 1:** Baseline characteristics of the study population in UK Biobank, by body mass index categories

	**Under- and normal weight **^**a**^ ***N* = 119,149 (35%)**	**Overweight **^**a**^ ***N* = 148,064 (43%)**	**Obesity **^**a**^ ***N* = 76,881 (22%)**	**Overall** ***N* = 344,094**
**Sex, n(%)**				
Female	76,055 (64)	66,409 (45)	38,764 (50)	181,228 (53)
**Age at assessment (years)**				
Median (IQR)	56.0 [48.0, 62.0]	57.0 [50.0, 63.0]	57.0 [50.0, 62.0]	57.0 [49.0, 62.0]
**Follow-up (years)**				
Median (IQR)	10.9 [10.1, 11.6]	10.9 [10.1, 11.7]	10.9 [10.1, 11.7]	10.9 [10.1, 11.7]
**Height (cm)**				
Median (IQR)	167 [162, 174]	170 [163, 177]	168 [161, 176]	169 [162, 176]
**Qualifications, n (%)**				
College or University degree	51,407 (43)	51,671 (35)	21,845 (28)	124,923 (36)
A levels/AS levels or equivalent	15,010 (13)	17,284 (12)	8,821 (11)	41,115 (12)
O levels/GCSEs or equivalent	23,953 (20)	32,021 (22)	17,602 (23)	73,576 (21)
CSEs or equivalent	5,493 (4.6)	7,874 (5.3)	4,998 (6.5)	18,365 (5.3)
NVQ or HND or HNC or equivalent	5,671 (4.8)	10,458 (7.1)	6,048 (7.9)	22,177 (6.4)
Other professional qualifications nursing, teaching	5,400 (4.5)	7,516 (5.1)	4,235 (5.5)	17,151 (5.0)
None of the above	12,215 (10)	21,240 (14)	13,332 (17)	46,787 (14)
**Smoking status, n (%)**				
Never	71,703 (60)	80,889 (55)	40,364 (52)	192,956 (56)
Previous	34,782 (29)	52,417 (35)	29,193 (38)	116,392 (34)
Current	12,664 (11)	14,758 (10)	7,324 (9.5)	34,746 (10)
**Alcohol consumption frequency, n (%)**				
Never	7,904 (6.6)	9,380 (6.3)	6,664 (8.7)	23,948 (7.0)
Special occasions only	11,080 (9.3)	13,650 (9.2)	10,889 (14)	35,619 (10)
One to three times a month	12,088 (10)	15,342 (10)	10,340 (13)	37,770 (11)
Once or twice a week	30,071 (25)	38,686 (26)	20,422 (27)	89,179 (26)
Three or four times a week	30,235 (25)	37,753 (25)	15,770 (20)	83,758 (24)
Daily or almost daily	27,771 (23)	33,253 (22)	12,796 (17)	73,820 (21)
**Diet score, n (%)**^**b**^				
6, healthier	4,452 (3.7)	3,511 (2.4)	1,447 (1.9)	9,410 (2.7)
5	27,652 (23)	27,909 (19)	12,587 (16)	68,148 (20)
4	51,532 (43)	62,650 (42)	30,876 (40)	145,058 (42)
3	26,883 (23)	40,009 (27)	22,763 (30)	89,655 (26)
2	7,108 (6.0)	11,476 (7.8)	7,377 (9.6)	25,961 (7.5)
1	1,339 (1.1)	2,224 (1.5)	1,624 (2.1)	5,187 (1.5)
0, unhealthier	183 (0.2)	285 (0.2)	207 (0.3)	675 (0.2)
**Physical activity, n (%)**				
**High**	53,248 (45)	61,193 (41)	26,220 (34)	140,661 (41)
Moderate	48,724 (41)	60,683 (41)	30,974 (40)	140,381 (41)
Low	17,177 (14)	26,188 (18)	19,687 (26)	63,052 (18)
**Menopause status, n (%)**^**c**^				
Premenopause	23,736 (31)	15,691 (24)	9,344 (24)	48,771 (27)
Postmenopause	43,180 (57)	40,668 (61)	22,283 (57)	106,131 (59)
Not sure	9,139 (12)	10,050 (15)	7,137 (18)	26,326 (14)
**Ever use HRT, n (%)**^**c**^				
No	51,133 (67)	40,401 (61)	24,462 (63)	115,996 (64)
Yes	24,922 (33)	26,008 (39)	14,302 (37)	65,232 (36)

Notes: more information on covariates, is given in Supplementary Table S2

*Abbreviations*: *UKB* UK Biobank, *IQR* Interquartile range, *HRT* hormone replacement therapy

^a^Following WHO categorisation of body mass index (BMI), Underweight [BMI < 18.5 kg/m^2^], Normal weight [BMI ≥ 18.5 and < 25 kg/m^2^], Overweight [BMI ≥ 25 and < 30 kg/m^2^], Obesity [BMI ≥ 30 kg/m^2^]

^b^Scores are arranged from the healthier (6)—Healthy diet score was calculated based on consumption of commonly eaten food groups following recommendations (see Supplementary Table S2)

^c^Only for women

**Table 2:** Baseline characteristics of the study population in EPIC, by body mass index categories

	**Under- and normal weight **^**a**^ ***n* = 102,010 (44%)**	**Overweight**^**a**^ ***n* = 94,878 (41%)**	**Obesity**^**a**^ ***n* = 36,361 (15%)**	**Overall**^**a**^ **(*n* = 233,249)**
**Sex, n (%)**				
Female	72,403 (71)	49,775 (52)	22,407 (62)	144,585 (62)
**Age at assessment (years)**				
Median (IQR)	51.5 [43.4, 57.3]	53.5 [47.5, 59.1]	53.7 [48.1, 59.4]	52.6 [46.0, 58.4]
**Follow-up (years)**				
Median (IQR)	10.6 [9.4, 11.8]	10.8 [9.5, 12.3]	11.2 [9.6, 12.8]	10.9 [9.4, 12.2]
**Height (cm)**				
Median (IQR)	166 [160, 173]	167 [160, 174]	164 [157, 171]	166 [160, 173]
**Qualifications, n (%)**				
Longer education (incl. University deg.)	26,186 (26)	17,582 (19)	4,246 (12)	48,014 (21)
Secondary school	17,760 (17)	11,628 (12)	3,296 (9.1)	32,684 (14)
Technical/professional school	30,084 (30)	25,326 (27)	8,361 (23)	63,771 (27)
Primary school completed	24,634 (24)	33,244 (35)	14,813 (41)	72,691 (31)
None	1,581 (1.5)	5,741 (6.1)	5,202 (14)	12,524 (5.4)
Not specified	1,765 (1.7)	1,357 (1.4)	443 (1.2)	3,565 (1.5)
**Smoking status, n (%)**				
Never	45,506 (45)	41,317 (44)	17,935 (49)	104,758 (45)
Previous	27,250 (27)	29,668 (31)	10,492 (29)	67,410 (29)
Current	29,254 (28)	23,893 (25)	7,934 (22)	61,081 (26)
**Alcohol consumption (g/day)**				
Median (IQR)	7.6 [1.5, 18.8]	8.7 [1.4, 23.5]	5.0 [0.4, 18.1]	7.6 [1.2, 20.5]
**Alcohol consumers status**				
Regular consumers	91,313 (90)	82,363 (87)	28,877 (79)	202,553 (87)
Non-consumers	10,697 (10)	12,515 (13)	7,484 (21)	30,696 (13)
**Mediterranean diet score, n (%)**^**b**^				
5—= < 6	1,975 (1.9)	2,330 (2.5)	1,005 (2.8)	5,310 (2.3)
4—< 5	15,669 (15)	16,074 (17)	6,680 (18)	38,423 (17)
3—< 4	34,108 (33)	30,866 (32)	12,082 (33)	77,056 (33)
2—< 3	34,263 (34)	30,200 (32)	10,741 (30)	75,204 (32)
1—< 2	14,501 (14)	13,932 (15)	5,207 (14)	33,640 (14)
0—< 1	1,494 (1.5)	1,476 (1.6)	646 (1.8)	3,616 (1.7)
**Physical activity, n (%)**				
Active	25,537 (25)	21,750 (23)	6,597 (18)	53,884 (23)
Moderately active	24,712 (24)	20,990 (22)	6,960 (19)	52,662 (23)
Moderately inactive	34,559 (34)	31,136 (33)	11,590 (32)	77,285 (33)
Inactive	17,202 (17)	21,002 (22)	11,214 (31)	49,418 (21)
**Ever use HRT, n (%)**^**c**^				
No	19,874 (89)	41,835 (84)	60,159 (83)	121,868 (84)
Yes	2,533 (11)	7,940 (16)	12,244 (17)	22,717 (16)
**Menopause status, n (%)**^**c**^				
Premenopausal	6,043 (27)	14,340 (29)	29,669 (41)	50,052 (35)
Postmenopausal	11,781 (53)	25,958 (52)	30,700 (42)	68,439 (47)
Perimenopause	3,364 (15)	7,199 (14)	10,031 (14)	20,594 (14)
Surgical menopause	1,219 (5.4)	2,278 (4.6)	2,003 (2.8)	5,500 (3.8)
**Country of recruitment**				
Denmark	22,953 (23)	21,621 (23)	7,267 (20)	51,841 (22)
Germany	19,505 (19)	17,548 (19)	6,685 (18)	43,738 (19)
Italy	20,095 (20)	17,528 (18)	5,955 (16)	43,578 (19)
Spain	8,180 (8.0)	17,411 (18)	10,361 (29)	35,952 (15)
The Netherlands	15,521 (15)	10,070 (11)	3,026 (8.3)	28,617 (12)
United Kingdom	15,756 (15)	10,700 (11)	3,067 (8.4)	29,523 (13)

Notes: more information on covariates is given in Supplementary Table S2

*Abbreviations*: *EPIC* European Prospective Investigation into Cancer and nutrition, *IQR* Interquartile range, *HRT* Hormone replacement therapy

^a^Following WHO categorisation of body mass index (BMI), Underweight [BMI < 18.5 kg/m^2^], Normal weight [BMI ≥ 18.5 and < 25 kg/m^2^], Overweight [BMI ≥ 25 and < 30 kg/m^2^], Obesity [BMI ≥ 30 kg/m^2^]

^b^The score is from the healthier (6 to 0)—18-point score which was combined in a 6-point score

^c^Only for women

### BMI and risk of obesity-related cancer by cardiometabolic disease status

**Fig. 1:**
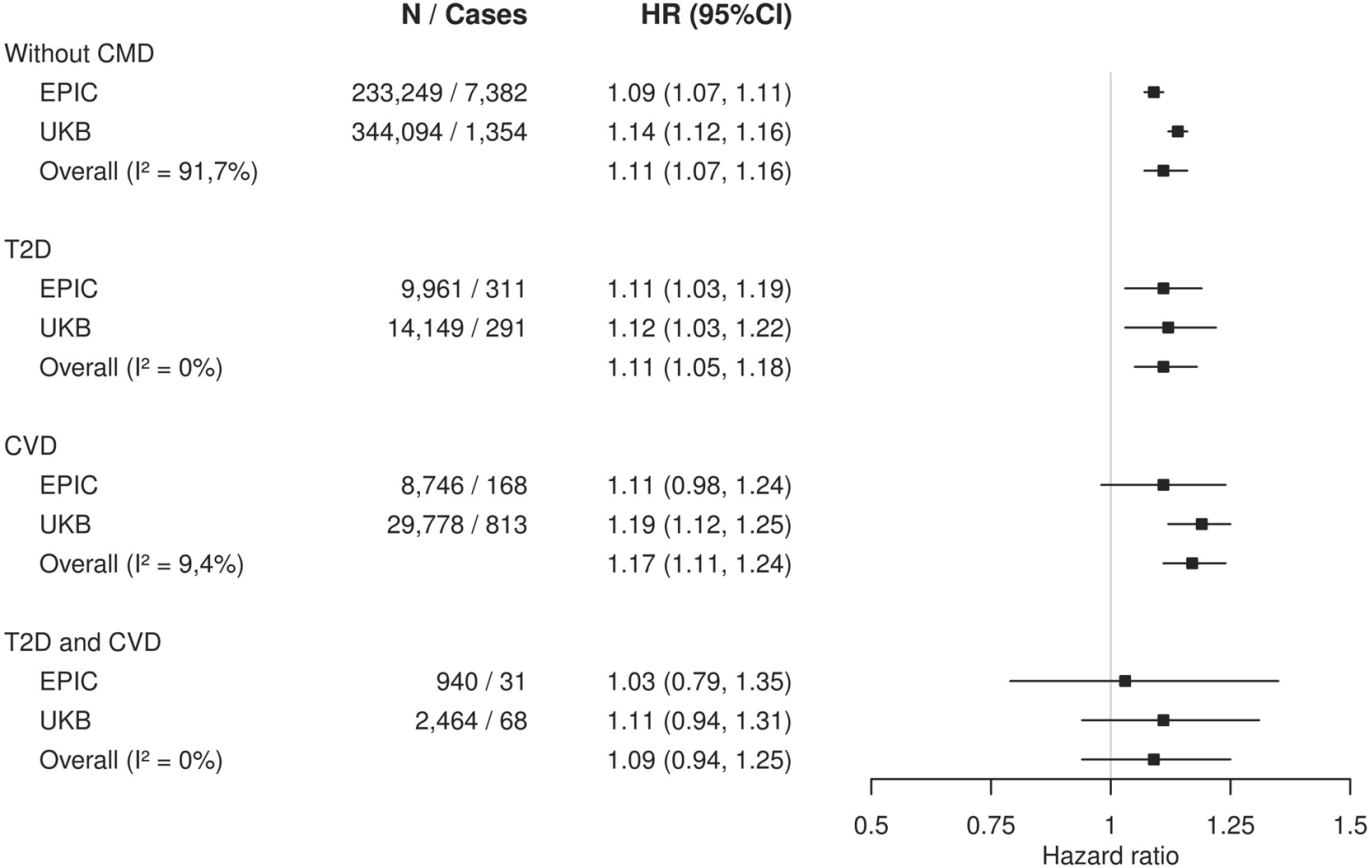
BMI and obesity-related cancer risk by cardiometabolic disease status in EPIC and UKB cohorts. *I*^2^, the proportion of total variability due to between-study heterogeneity from random effects meta-analysis. BMI body mass index, EPIC European Prospective Investigation into Cancer and nutrition, UKB UK Biobank, HR hazard ratio, CI confidence interval, T2D type 2 diabetes, CVD cardiovascular diseases, CMD cardiometabolic diseases

In our multivariable-adjusted base model, where we ignored CMD status, an expected positive association was observed between BMI and obesity-related cancer risk. Per 1 SD increment in BMI (~ 5 kg/m^2^), HRs were 1.10 (95% CI 1.08–1.12) and 1.16 (95% CI: 1.14–1.18) in EPIC and UKB, respectively (Table 3). Adjustment for CMD and its duration slightly attenuated these associations (Table 3). In our main model, we estimated associations by CMD status. In the meta-analysis of both cohorts, BMI, per 1 SD increment, was positively associated with the risk of obesity-related cancers among participants without CMD (summary HR: 1.11, 95% CI: 1.07–1.16) and, similarly, among participants with T2D or CVD (Fig. 1) with little evidence for multiplicative interaction (all *P*-interaction ≥ 0.17) (Table 3). The strongest positive association was observed among participants with CVD in the UKB with a HR equal to 1.19 (95% CI: 1.12–1.25) (Fig. 1 and Table 3). The high proportion of total variability attributed to between-study heterogeneity (*I*^2^ = 92%) for models without CMD (Fig. 1) is likely due to a combination of the precise risk estimates and the different prevalence of obesity among participants in the UK Biobank (22%) as compared to EPIC (15%). Results did not materially differ between men and women (Additional File 1: Figures S5 and S6, Tables S3 and S4) except those for CVD, where we observed a stronger association between BMI- and obesity-related cancer risk among women than men (summary HR women: 1.22, 95% CI: 1.13–1.31; summary HR men: 1.07, 95% CI: 0.96–1.19).

**Table 3:** Association between BMI (per 1 standard deviation increment^1^) and the risk of obesity-related cancer by ascertainment of cardiometabolic diseases, in EPIC and UKB cohorts

	**EPIC**					**UKB**				
	**N at risk / N cases**	**HR (95% CI)**	**p**^**2**^	**p**^**3**^	**p**^**4**^	**N at risk / N cases**	**HR (95% CI)**	**p**^**2**^	**p**^**3**^	**p**^**4**^
**Obesity-related cancers**										
**Base model**^**5**^	233,249 / 7,892	1.10 (1.08; 1.12)	< 0.001			344,094 / 12,526	1.16 (1.14; 1.18)	< 0.001		
**Overall adj. model**^**6**^	233,249 / 7,892	1.09 (1.07; 1.11)	< 0.001			344,094 / 12,526	1.14 (1.12; 1.16)	< 0.001		
**Without CMD**^**7,8**^	233,249 / 7,382	1.09 (1.07; 1.11)	< 0.001		0.95	344,094 / 1,354	1.14 (1.12; 1.16)	< 0.001		0.52
**T2D**^**7**^	9,961 / 311	1.11 (1.03; 1.19)	0.005	0.75		14,149 / 291	1.12 (1.03; 1.22)	< 0.001	0.68	
**CVD**^**7**^	8,746 / 168	1.11 (0.98; 1.24)	0.09	0.84		29,778 / 813	1.19 (1.12; 1.25)	< 0.001	0.17	
**T2D-CVD**^**7**^	940 / 31	1.03 (0.79; 1.35)	0.81	0.67		2,464 / 68	1.11 (0.94;1.31)	0.23	0.74	

*Abbreviations*: *SD* standard deviation, *BMI* body mass index, *HR* hazard ratio, *95%CI* 95% confidence intervals, *T2D* type 2 diabetes, *CVD* cardiovascular diseases, *CMD* cardiometabolic diseases, *EPIC* European Prospective Investigation into Cancer and nutrition, *UKB* UK Biobank

^1^Standard deviation (SD) of BMI in EPIC = 4.18 kg/m^2^; Standard deviation (SD) of BMI in UKB = 4.60 kg/m^2^

*P-values: *^2^ *p*-values for association of local association tests;^3^ *p*-values for the individual interaction term; ^4^ *p*-value from likelihood ratio test comparing models with and without an interaction term

^5^Base model adjusted for educational level, alcohol consumption, smoking status, height, physical activity, healthy diet score, menopausal status for women, use of HRT for women, and stratified by centre of recruitment, age (5-year categories), and sex

^6^Overall adjusted model (further adjusted for CMD, modelled as a time-varying variable, and the duration of these comorbidities)

^7^Overall adjusted model with an interaction term between BMI and CMD (modelled as a time-varying variable)

^8^Without CVD and T2D

In sensitivity analyses, further adjustments for the use of metformin or statins in the UKB or analyses among never smokers resulted in risk estimates similar to our main models (Additional File 1: Tables S5, S6, and S7). We also assessed associations between BMI and the risk of non-obesity related cancers as a negative control outcome and risk estimates were largely null (Additional File 1: Figures S5, S6 and S7, Tables S5, S7, and S8).

### Joint association of overweight and/or obesity and cardiometabolic diseases with obesity-related cancer risk

**Fig. 2:**
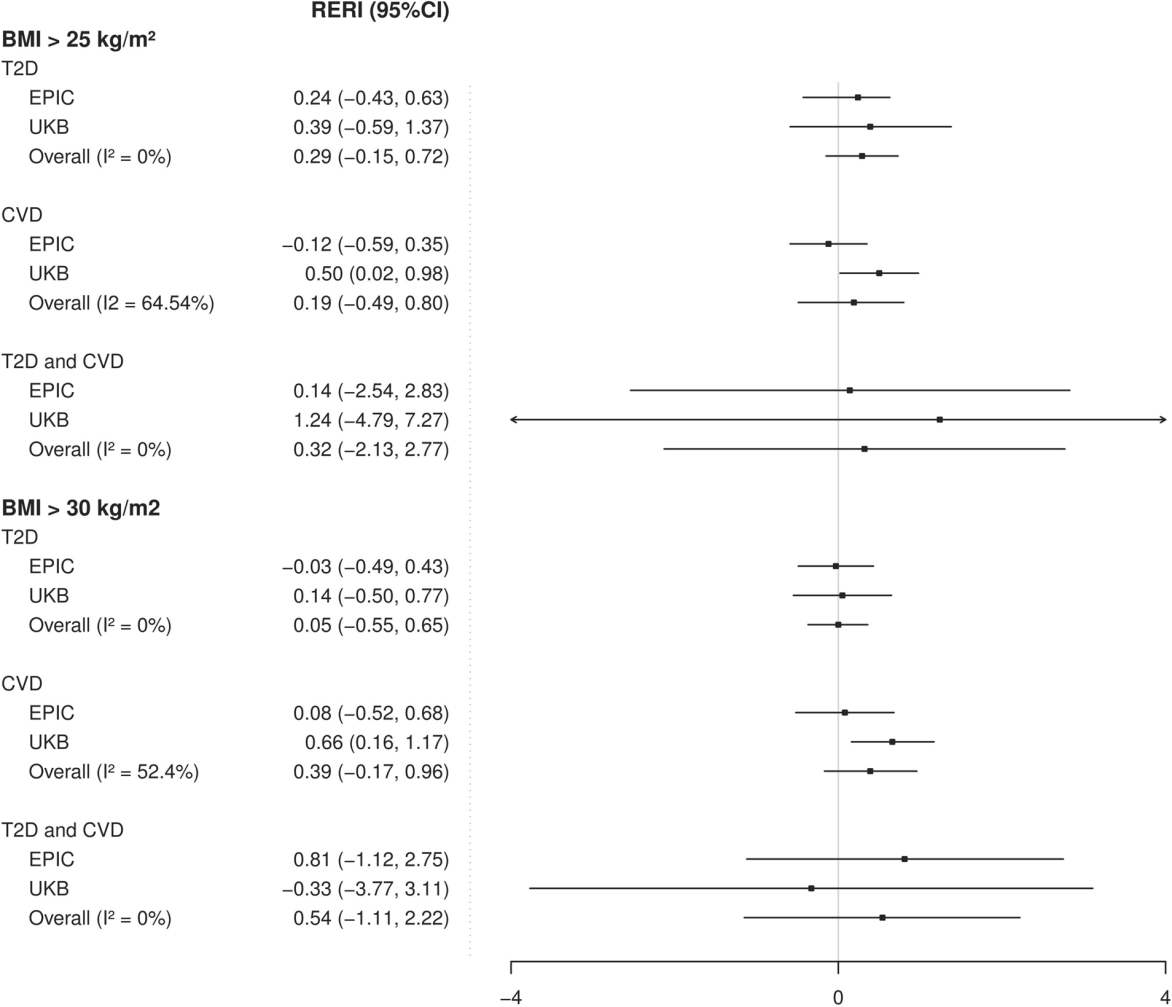
Relative excess risk of obesity-related cancers due to interaction between overweight/obesity and cardiometabolic diseases in EPIC and UKB cohorts. *I*^2^, the proportion of total variability due to between-study heterogeneity from random effects meta-analysis. CI confidence interval, EPIC European Prospective Investigation into Cancer and nutrition, UKB UK Biobank, RERI relative excess risk due to interaction, BMI body mass index, T2D type 2 diabetes, CVD cardiovascular diseases

The separate and joint associations of overweight/obesity and CMD with obesity-related cancer risk are shown in Table 4. In the UKB, there was evidence that the joint association of overweight and CVD was higher than the sum of the separate associations. Compared to participants with normal weight (BMI < 25 kg/m^2^) and without a CVD (reference group), participants with both exposures (BMI ≥ 25 kg/m^2^ and a CVD) had a 3.4 times higher risk of obesity-related cancer (95% CI: 2.95–3.95). Respective HR for participants with normal weight, but with a CVD, and for participants with overweight/obesity without CVD were 2.68 (95% CI 2.23–3.23) and 1.23 (95% CI 1.18–1.28) (Table 4). This additive interaction corresponded to a RERI of 0.50 (95% CI: 0.02–0.98), meaning that the joint association was 0.5 times higher than the sum of the separate associations. The joint association of obesity (BMI ≥ 30 kg/m^2^) and CVD resulted in a slightly stronger RERI (0.66, 95% CI: 0.16–1.17). However, these interactions were not confirmed in EPIC (Table 4) and the CI of the RERI in the meta-analysis of both cohorts included the null; for example, the meta-analysed RERI of obesity (BMI ≥ 30 kg/m^2^) and CVD was 0.39 (95% CI − 0.17–0.96) (Fig. 2). In sex-stratified analysis, the RERI included the null among men (Additional File 1: Figure S8 and Table S9) while in women, the joint association of obesity and CVD was more than additive (summary RERI: 0.90 95% CI: 0.34–1.45) (Additional File 1: Figure S9 and Table S10).

**Table 4:** Relative excess risk of obesity-related cancers due to interaction between overweight and/or obesity (BMI ≥ 25 kg/m^2^ or BMI ≥ 30 kg/m^2^) and cardiometabolic diseases, in EPIC and UKB cohorts

	**EPIC**						**UKB**					
	**T2D**		**CVD**		**T2D and CVD**		**T2D**		**CVD**		**T2D and CVD**	
	**N at risk / N cases**	**HR (95% CI)**	**N at risk / N cases**	**HR (95% CI)**	**N at risk / N cases**	**HR (95% CI)**	**N at risk / N cases**	**HR (95% CI)**	**N at risk / N cases**	**HR (95% CI)**	**N at risk / N cases**	**HR (95% CI)**
**BMI < 25 kg/m**^**2**^**, without CMD of interest**^**a**^	100,714 / 3,150	1.00 (ref)	99,096 / 3,127	1.00 (ref)	101,886 / 3,185	1.00 (ref)	177,892 / 3,859	1.00 (ref)	111,433 / 3,685	1.00 (ref)	118,934 / 3,878	1.00 (ref)
**BMI < 25 kg/m**^**2**^**, with CMD**	1,296 / 38	1.87 (1.25; 2.81)	2,914/61	1.47 (0.98; 2.19)	124 / 3	2.09 (0.58; 7.48)	1,257 / 24	2.28 (1.44; 3.60)	7,716 / 188	2.68 (2.23; 3.23)	215 /5	6.45 (2.58; 16.16)
**BMI ≥ 25 kg/m**^**2**^**, without CMD of interest**	122,574 / 4,431	1.12 (1.07; 1.17)	125,407 / 4,597	1.12 (1.07; 1.18)	130,423 / 4,676	1.12 (1.07; 1.17)	213,352 / 8,376	1.23 (1.18; 1.28)	204,048 / 8,018	1.23 (1.18; 1.28)	222,696 / 8,580	1.23 (1.18; 1.28)
**BMI ≥ 25 kg/m**^**2**^**, with CMD (joint effect)**	8,665 / 273	2.23 (1.68; 2.95)	5,832 / 107	1.47 (0.98; 2.13)	816 / 28	2.35 (1.25; 4.44)	11,593 / 267	2.90 (2.25; 3.74)	20,897 / 625	3.41 (2.95; 3.95)	2,249 / 63	7.93 (5.49; 11.44)
**RERI**		**0.24 (-0.43; 0.63)**		**-0.12 (-0.59; 0.35)**		**0.14 (-2.54; 2.83)**		**0.39 (-0.59; 1.38)**		**0.50 (0.02; 0.98)**		**1.24 (-4.79; 7.27)**
**BMI < 30 kg/m**^**2**^**, without CMD of interest**^**a**^	191,346 / 6,252	1.00 (ref)	189,779 / 6,279	1.00 (ref)	196,344 / 6,399	1.00 (ref)	261,442 / 9,150	1.00 (ref)	246,173 / 8,730	1.00 (ref)	266,132 / 9,249	1.00 (ref)
**BMI < 30 kg/m**^**2**^**, with CMD**	5,542 / 164	2.28 (1.81; 2.88)	7,109 / 137	1.48 (1.09; 2.01)	544 / 17	2.93 (1.72; 5.00)	5,771 / 127	2.44 (1.84; 3.23)	21,040 / 547	2.69 (2.32; 3.10)	1,081 /28	7.14 (4.53; 11 27)
**BMI ≥ 30 kg/m**^**2**^**, without CMD of interest**	31,942 / 1,329	1.19 (1.12; 1.26)	34,724 / 1,445	1.18 (1.11; 1.24)	35,965 / 1,462	1.18 (1.11; 1.24)	69,802 / 3,085	1.24 (1.19; 1.30)	69,308 / 2,983	1.23 (1.18; 1.29)	75,498 / 3,209	1.24 (1.19; 1.29)
**BMI ≥ 30 kg/m**^**2**^**, with CMD (joint effect)**	4,419 / 147	2.44 (1.91; 3.12)	1,637 / 31	1.74 (1.15; 2.61)	396 / 14	1.17 (1.11; 1.24)	7,076 / 164	2.73 (2.09; 3.57)	7,573 / 266	3.58 (3.02; 4.24)	1,383 / 40	7.05 (4.66; 10.66)
**RERI**		**-0.03 (-0.49; 0.43)**		**0.08 (-0.52; 0.68)**		**0.81 (-1.12; 2.75)**		**0.05 (-0.55; 0.65)**		**0.66 (0.16; 1.17)**		**-0.33 (-3.77; 3.11)**

Notes: The model included BMI as a binary variable with an interaction term with the binary variable of the existence of the disease of interest and was adjusted for duration of comorbidities, educational level, smoking status, alcohol consumption, height, use of HRT, menopausal status, physical activity, healthy diet score, two binary variables of the two cardiometabolic conditions status not studied and stratified by sex, centre, and age at recruitment (5-year categories)

RERI_RR_ = RR_11_ − RR_10_ − RR_01_ + 1, where _11_ denotes being exposed to both factors (e.g., overweight/obesity and T2D), _10_ to one factor (e.g., overweight/obesity), and _01_ to the other one (e.g., *T2D*). A RERI of 0 was considered a lack of additive interaction and 95% CIs were calculated as proposed by *Hosmer and Lemeshow*

*Abbreviations*: *BMI* body mass index, *T2D* type 2 diabetes, *CVD* cardiovascular diseases, *HR* hazard ratio, *95%CI* 95% confidence intervals, *RERI* relative excessive risk due to interaction, *CMD* cardiometabolic diseases, *EPIC* European Prospective Investigation into Cancer and nutrition, *UKB* UK Biobank

^a^Without diseases mean without the combination of the disease(s) studied

In sensitivity analyses of the joint effect, further adjustments for the use of metformin or statins did not alter the results while analyses among never smokers in UKB showed a higher positive additive interaction for the joint association of obesity and CVD (RERI: 0.83, 95% CI: 0.03–1.64) (Additional File 1: Table S11).

### BMI and risk of total cancer by cardiometabolic disease status

In our multivariable-adjusted base model, ignoring CMD status, BMI (per 1 SD) was positively associated with overall cancer risk with HRs of 1.04 (95% CI 1.02–1.05) and 1.05 (95% CI: 1.04–1.06) in EPIC and UKB, respectively (Additional File 1: Table S3). Adjustment for CMD and its duration did not change these associations (Additional File 1: Table S3). After stratification by CMD, in the meta-analysis of both cohorts, BMI (per 1 SD increment) was positively associated with the risk of overall cancer among participants without CMD (summary HR: 1.04, 95% CI: 1.03–1.05) and among those with CVD (the summary HR: 1.05, 95% CI: 1.01–1.08). Among individuals with T2D, or with both T2D and CVD, associations were largely null (Additional File 1: Figure S7). Associations across categories of CMD were stronger among women as compared to men. A positive association among women without CMD (summary HR: 1.06, 95% CI: 1.02–1.10), with T2D (summary HR: 1.09, 95% CI: 1.00–1.19) and with CVD (summary HR: 1.13, 95% CI: 1.07–1.19) was found whereas associations were null among men (Additional File 1: Figures S5 and S6).

In sensitivity analyses, we observed that the associations were similar in UKB when further adjusted for metformin and statins use (Additional File 1: Tables S5 and S6). The associations among never-smokers tended to be slightly stronger than those among the total population (Additional File 1: Table S7).

### Joint association of overweight and/or obesity and cardiometabolic diseases with total cancer risk

The separate and joint associations of overweight/obesity and CMD with overall cancer risk are shown in Additional File 1: Table S12. Compared to participants with normal weight (BMI < 25 kg/m^2^) and without a CVD (reference group), participants with obesity (BMI ≥ 30 kg/m^2^) and a CVD had a 2 times higher risk of overall cancer (95% CI: 1.62–2.51) in the EPIC study. Similar estimates were obtained in the UKB (HR 2.59, 95% CI: 2.32–2.88). In the meta-analysis of both cohorts, these joint associations of obesity and CVD translated into a summary RERI of 0.28 (95% CI: 0.09–0.47) (Additional File 1: Figure S10). Among participants with overweight/obesity and CVD, the joint association was attenuated (summary RERI: 0.11, 95% CI: − 0.07–0.29) (Additional File 1: Figure S10). In sex-stratified analysis, the RERI included the null among men (Additional File 1: Figure S8) while in women, the joint association of obesity and CVD was more than additive (summary RERI: 0.75, 95% CI: 0.35–1.16) as well as the joint association of overweight/obesity and T2D (summary RERI: 0.41, 95% CI: 0.02–0.81) (Additional File 1: Figure S9).

In sensitivity analyses of the joint effect, further adjustments for the use of metformin or statins did not alter the results (Additional File 1: Tables S13 and S14), while analyses among never smokers in UKB showed a positive additive interaction for the joint association of overweight/obesity and CVD and for obesity and CVD (respectively, RERI: 0.35, 95% CI: 0.01–0.70 and RERI: 0.45, 95% CI: 0.07–0.83) (Additional File 1: Table S11).

## Discussion

In this meta-analysis of two of the largest European prospective cohorts with together almost 600,000 adult participants, we found that the joint association of obesity and CVD with cancer risk was greater than the sum of the separate associations of the two exposures with cancer risk. This finding was consistent across the two cohorts for overall cancer risk but was only observed in the UKB for obesity-related cancer risk. Provided that the findings of an additive interaction are confirmed in subsequent studies, this implies that obesity prevention would translate into a greater cancer risk reduction among population groups with CVD than among the general population.

While a high BMI, which is indicative of overweight or obesity, is a recognised risk factor for certain cancers in the general population [3, 37–39], the differences in cancer risk among population groups affected by CMD remained poorly understood [18]. We addressed this knowledge gap and showed that a high BMI was equally associated with an increased risk of obesity-related cancer in population groups with and without a CMD. Our findings were similar for the risk of total cancer, except that in the population group with T2D only, BMI was not associated with the risk of total cancer. This contrasts with a meta-analysis of six cohort studies, where BMI was positively associated with the risk of total cancer among T2D patients [19]. A possible explanation for this discrepancy is that the meta-analysis included cohorts from North America and Asia with likely differences in the proportion of cancer types included in the composite outcome of total cancer, and that individuals with diabetes at baseline were included, thus BMI was assessed after diabetes diagnosis.

We provide novel evidence for an additive interaction between overweight/obesity and a concurrent CVD in relation to overall and obesity-related cancer risk. This is supported by the findings of our companion study using real-world data, where a similar additive interaction between overweight/obesity and a CVD was observed in relation to obesity-related cancer risk (overall cancer was not investigated) [16]. In the context of a rising prevalence of multimorbidity [22], i.e., multiple diseases affecting individuals and groups, it is important to understand why certain diseases cluster and whether or to what degree their clustering impacts the disease burden of affected populations. This emergent approach to health and clinical practice has been described as the syndemics model of health [40]. Syndemics provides a framework for empirically evaluating how disease clustering arises in a population and what health interventions might be most effective for mitigating them [40].

Excess body weight is a risk factor common to CMD and cancer, which is one likely reason for the clustering of these diseases. Different biological mechanisms are proposed to explain the adiposity-cancer link including altered sex hormone metabolism, increased insulin levels and the bioavailability of insulin-like growth factor 1 [IGF1], adipokine pathophysiology, and systemic inflammation [37]. T2D, an established risk factor for many cancers that are obesity-related [7–12], likely influences cancer risk through mechanisms similar to obesity [6, 39]. In contrast, the CVD–cancer relationship is only emerging. A seminal study demonstrated in a mouse model that heart failure due to myocardial infarction resulted in increased intestinal tumor growth [13]. Secreted cardiac proteins into the bloodstream, in particular SerpinA3, after cardiac injury were identified as the cause of this phenomenon [13]. A subsequent in vivo study in mice supported cardiac remodeling as an acute pathologic stressor that induced breast and lung cancer growth by the secretion of protumorigenic factors [41]. Moreover, in a breast cancer model, myocardial infarction induced a systemic host response toward an immunosuppressive state that accelerated breast cancer growth [42]. Taken together, CVD can promote growth of certain cancers in the experimental setting and might also do so in humans.

Our study has several strengths. First, we meta-analysed individual-level participant data from two large prospective cohorts of adults across 6 European countries with validated assessments of cancer, CVD, and T2D. Second, to our knowledge, this is the largest study using multinational data to investigate the association between BMI and cancer incidence by ascertainment of CMD. Third, associations were modelled in a time-varying fashion accounting for the sequence of incident chronic conditions. Fourth, we performed sensitivity analyses to address potential biases.

Our findings need to be interpreted considering the following limitations. First, information on lifestyle habits and BMI assessed at recruitment for both cohorts were used while possible changes in modifiable habits during follow-up could not be considered. However, there is evidence that a large proportion of individuals diagnosed with a new onset chronic condition did not change their lifestyle habits [43]. Additionally reassuring are the results of our companion study, where the cancer risk estimates based on baseline BMI were very similar to those when updated BMI assessments during follow-up were used [16]. Second, metformin, a first line medication for T2D, may lead to weight loss and may also reduce the risk of certain cancers [44, 45]. While we could account for metformin use, and statin use, in the UKB, such data are not available in EPIC. However, in the UKB, adjustment for CMD treatment did not alter our findings. Third, selection bias, due to excluding participants with missing data and due to restricting analyses to adults without a CMD at recruitment, could have influenced our results. Moreover, a major limitation of the UK Biobank cohort is its low response rate to recruitment invitations (~ 5%), significantly lower compared to many cohorts [46, 47]. The participants have been observed to be less socioeconomically deprived, have fewer risk factors, and have a lower prevalence of long-term conditions compared to the general UK population [48]. We partially addressed this concern of selection bias by providing estimates for a negative control outcome (i.e., non-obesity related cancers). Respective associations were largely null, supporting our main findings. Fourth, BMI neither captures body fat distribution nor differentiates between lean mass and fat mass. Future studies using detailed body composition assessments (e.g., from magnetic resonance imaging) could provide insight into the role of different fat compartments in the cardiometabolic disease–cancer relationship. Last, recruitment into the two cohorts varied between 1992 and 2000 for EPIC and between 2006 and 2010 for UKB. This temporal discrepancy may explain some of the observed inconsistencies in the strength of the associations between the two cohorts, as changes in lifestyle or in treatment and management of CMD could have occurred during this period.

## Conclusions

In these two large cohorts, higher BMI was associated with an increased risk of obesity-related cancer among European adults irrespective of their CMD status. However, the joint exposure to overweight/obesity and CVD may be associated with a higher risk of overall and obesity-related cancer than the sum of the separate associations of the two exposures. These findings suggest that obesity prevention in population groups with CVD would lead to a greater reduction in cancer incidence as compared to the general population. Further studies that investigate associations of adiposity with the incidence of specific cancer sites among population groups with T2D or CVD are warranted.

### Supplementary Information

**Additional file 1: Figure S1:** Flowchart with the inclusion and exclusion criteria of the study population, in UK Biobank; **Figure S2:** Flowchart with the inclusion and exclusion criteria of the study population, in EPIC; **Table S1:** Diagnostic codes for the definition of cancer, type 2 diabetes and cardiovascular diseases; **Table S2:** Main characteristics of both cohorts; **Figure S3:** Directed Acyclic Graph (DAG) describing the potential causal and confounding effects of obesity on cancer risk; **Figure S4:** Framework to study the natural history of multimorbidity in UKB and EPIC with cancer as the index disease and T2D and CVD as comorbidities; **Table S3:** Association between BMI (per 1 standard deviation increment) and cancer risk by ascertainment of incident cardiometabolic conditions, in EPIC and UKB cohorts, in men; **Figure S5:** Forest plot of the associations (with 95% CIs) between BMI and cancer risk depending on cardiometabolic status in EPIC and UKB cohorts and the results of the meta-analysis (random-effects models), in men; **Table S4:** Association between BMI (per 1 standard deviation increment) and cancer risk by ascertainment of incident cardiometabolic conditions, in EPIC and UKB cohorts, in women; **Figure S6:** Forest plot of the associations (with 95% CIs) between BMI and cancer risk depending on cardiometabolic status in EPIC and UKB cohorts and the results of the meta-analysis (random-effects models), in women; **Table S5:** Association between BMI (per 1 standard deviation increment) and cancer risk by ascertainment of incident cardiometabolic conditions, with 95% CIs, in UKB. Models further adjusted for metformin use; **Table S6:** Association between body mass index (per 1 standard deviation increment) and cancer risk by ascertainment of incident cardiometabolic conditions, with 95% CIs, in UKB. Models further adjusted for statin use; **Table S7:** Association between BMI (per 1 standard deviation increment) and cancer risk by ascertainment of incident cardiometabolic conditions, in EPIC and UKB cohorts, among never smokers; **Table S8:** Association between BMI (per 1 standard deviation increment) and cancer risk (total cancers and non-obesity-related cancers) by ascertainment of incident cardiometabolic conditions, in EPIC and UKB cohorts; **Figure S7:** Forest plot of the associations (with 95% CIs) between BMI and cancer risk (total cancers and non-obesity-related cancers) by ascertainment of incident cardiometabolic conditions, in EPIC and UKB cohorts and the results of the meta-analysis (random-effects models); **Table S9:** Relative excess risk of all-cancers due to interaction between overweight and/or obesity and incident cardiometabolic diseases, in EPIC and UKB cohorts, in men; **Figure S8:** Forest plot of the relative excess risk of all-cancers due to interaction (with 95% CIs) between overweight and/or obesity and incident cardiometabolic diseases in EPIC and UKB cohorts and the results of the meta-analysis (random-effects models), in men; **Table S10:** Relative excess risk of all-cancers due to interaction between overweight and/or obesity and incident cardiometabolic diseases, in EPIC and UKB cohorts, in women; **Figure S9:** Forest plot of the relative excess risk of all-cancers due to interaction (with 95% CIs) between overweight and/or obesity and incident cardiometabolic diseases in EPIC and UKB cohorts and the results of the meta-analysis (random-effects models), in women; **Table S11:** Relative excess risk of all-cancers due to interaction between overweight and/or obesity and incident cardiometabolic diseases, in EPIC and UKB cohorts, among never smokers; **Figure S10:** Forest plot of the relative excess risk of all-cancers due to interaction (with 95% CIs) between overweight and/or obesity and incident cardiometabolic diseases, in EPIC and UKB cohorts and the results of the meta-analysis (random-effects models); **Table S12:** Relative excess risk of all-cancers due to interaction between overweight and/or obesity and incident cardiometabolic diseases, in EPIC and UKB cohorts; **Table S13:** Relative excess risk of all-cancers due to interaction between overweight and/or obesity and incident cardiometabolic diseases, in EPIC and UKB cohorts. Models further adjusted for metformin use; **Table S14:** Relative excess risk of all-cancers due to interaction between overweight and/or obesity and incident cardiometabolic diseases, in EPIC and UKB cohorts. Models further adjusted for statin use.

## Data Availability

The UK Biobank resource is available to bona fide researchers for health-related research in the public interest. All researchers who wish to access the research resource must register with UK Biobank by completing the registration form in the Access Management System (AMS- https://bbams.ndph.ox.ac.uk/ams/). EPIC data are available for investigators who seek to answer important questions on health and disease in the context of research projects that are consistent with the legal and ethical standard practices of IARC/WHO and the EPIC Centres. For information on how to submit an application for gaining access to EPIC data and/or biospecimens, please follow the instructions http://epic.iarc.fr/access/index.php. The datasets used and/or analyzed during the current study were accessed in 2021 and are available from the corresponding author on reasonable request.

## Abbreviations

BMI: Body mass index
CI: Confidence interval
CMD: Cardiometabolic diseases
CVD: Cardiovascular disease
EPIC: European Prospective Investigation into Cancer and nutrition
HR: Hazard ratio
NCD: Noncommunicable diseases
RERI: Relative excess risk due to interaction
SD: Standard deviation
T2D: Type 2 diabetes
UKB: UK Biobank
WHO: World Health Organization
