# Supplementary material for: Body mass index and cancer risk among adults with and without cardiometabolic diseases: evidence from the EPIC and UK Biobank prospective cohort studies
Source: BMC Med. 2023 Nov 23;21:418. doi: 10.1186/s12916-023-03114-z (PMC10666332; doi:10.1186/s12916-023-03114-z)
Supplement: Supplementary file 1 — Additional file 1: Figure S1: Flowchart with the inclusion and exclusion criteria of the study population, in UK Biobank; Figure S2: Flowchart with the inclusion and exclusion criteria of the study population, in EPIC; Table S1: Diagnostic codes for the definition of cancer, type 2 diabetes and cardiovascular diseases; Table S2: Main characteristics of both cohorts; Figure S3: Directed Acyclic Graph (DAG) describing the potential causal and confounding effects of obesity on cancer risk; Figure S4: Framework to study the natural history of multimorbidity in UKB and EPIC with cancer as the index disease and T2D and CVD as comorbidities; Table S3: Association between BMI (per 1 standard deviation increment) and cancer risk by ascertainment of incident cardiometabolic conditions, in EPIC and UKB cohorts, in men; Figure S5: Forest plot of the associations (with 95% CIs) between BMI and cancer risk depending on cardiometabolic status in EPIC and UKB cohorts and the results of the meta-analysis (random-effects models), in men; Table S4: Association between BMI (per 1 standard deviation increment) and cancer risk by ascertainment of incident cardiometabolic conditions, in EPIC and UKB cohorts, in women; Figure S6: Forest plot of the associations (with 95% CIs) between BMI and cancer risk depending on cardiometabolic status in EPIC and UKB cohorts and the results of the meta-analysis (random-effects models), in women; Table S5: Association between BMI (per 1 standard deviation increment) and cancer risk by ascertainment of incident cardiometabolic conditions, with 95% CIs, in UKB. Models further adjusted for metformin use; Table S6: Association between body mass index (per 1 standard deviation increment) and cancer risk by ascertainment of incident cardiometabolic conditions, with 95% CIs, in UKB. Models further adjusted for statin use; Table S7: Association between BMI (per 1 standard deviation increment) and cancer risk by ascertainment of incident cardiometabolic conditio [file 12916_2023_3114_MOESM1_ESM.docx]

**Body mass index and cancer risk among adults with and without cardiometabolic diseases: evidence from the EPIC and UK Biobank prospective cohort studies.**

**Additional File 1:**

[Figure S1: Flowchart with the inclusion and exclusion criteria of the study population, in UK Biobank 4](#_Toc147753114)

[Figure S2: Flowchart with the inclusion and exclusion criteria of the study population, in EPIC 6](#_Toc147753115)

[Table S1: Diagnostic codes for the definition of cancer, Type 2 Diabetes and cardiovascular diseases 7](#_Toc147753116)

[Table S2: Main design characteristics of both cohorts 9](#_Toc147753117)

[Figure S3: Directed Acyclic Graph (DAG) describing the potential causal and confounding effects of obesity on cancer risk 12](#_Toc147753118)

[Figure S4: Framework to study the natural history of multimorbidity in UKB and EPIC with cancer as index disease and T2D and CVD as comorbidities 13](#_Toc147753119)

[Table S3: Association between BMI (per 1 standard deviation increment^7^) and cancer risk by ascertainment of incident cardiometabolic conditions, in EPIC and UKB cohorts, in men 14](#_Toc147753120)

[Figure S5: Forest plot of the associations (with 95% Cis) between BMI and cancer risk depending on cardiometabolic status in EPIC and UKB cohort and the results of the meta-analysis (random effect models), in men 15](#_Toc147753121)

[Table S4: Association between BMI (per 1 standard deviation increment^7^) and cancer risk by ascertainment of incident cardiometabolic conditions, in EPIC and UKB cohorts, in women 16](#_Toc147753122)

[Figure S6: Forest plot of the associations (with 95% CIs) between BMI and cancer risk depending on cardiometabolic status in EPIC and UKB cohort and the results of the meta-analysis (random effect models), in women 17](#_Toc147753123)

[Table S5: Association between BMI (per 1 standard deviation increment^7^) and cancer risk by ascertainment of incident cardiometabolic conditions, with 95% Cis, in UKB. Models further adjusted on metformin use 18](#_Toc147753124)

[Table S6: Association between body mass index (per one standard deviation increment^7^) and cancer risk by ascertainment of incident cardiometabolic conditions, with 95% CIs, in UKB. Models further adjusted on statins use 19](#_Toc147753125)

[Table S7: Association between BMI (per 1 standard deviation increment^7^) and cancer risk by ascertainment of incident cardiometabolic conditions, in EPIC and UKB cohorts, among never smokers 20](#_Toc147753126)

[Table S8: Association between BMI (per 1 standard deviation increment^7^) and cancer risk (total cancers and non-obesity-related cancers) by ascertainment of incident cardiometabolic conditions, in EPIC and UKB cohorts 21](#_Toc147753127)

[Figure S7: Forest plot of the associations (with 95% CIs) between BMI and cancer risk (total cancers and non-obesity-related cancers) by ascertainment of incident cardiometabolic conditions, in EPIC and UKB cohort and the results of the meta-analysis (random effect models) 22](#_Toc147753128)

[Table S9: Relative excess risk of all-cancers due to interaction between overweight and/or obesity and incident cardiometabolic diseases, in EPIC and UKB cohorts, in men. 23](#_Toc147753129)

[Figure S8: Forest plot of the relative excess risk of all-cancers due to interaction (with 95% CIs) between overweight and/or obesity and incident cardiometabolic diseases in EPIC and UKB cohort and the results of the meta-analysis (random effect models), in men 24](#_Toc147753130)

[Table S10: Relative excess risk of all-cancers due to interaction between overweight and/or obesity and incident cardiometabolic diseases, in EPIC and UKB cohorts, in women. 25](#_Toc147753131)

[Figure S9: Forest plot of the relative excess risk of all-cancers due to interaction (with 95% Cis) between overweight and/or obesity and incident cardiometabolic diseases in EPIC and UKB cohort and the results of the meta-analysis (random effect models), in women 26](#_Toc147753132)

[Table S11: Relative excess risk of all-cancers due to interaction between overweight and/or obesity and incident cardiometabolic diseases, in EPIC and UKB cohorts, among never smokers. 27](#_Toc147753133)

[Figure S10: Forest plot of the relative excess risk of all-cancers due to interaction (with 95% CIs) between overweight and/or obesity and incident cardiometabolic diseases, in EPIC and UKB cohorts and the results of the meta-analysis (random effect models) 28](#_Toc147753134)

[Table S12: Relative excess risk of all-cancers due to interaction between overweight and/or obesity and incident cardiometabolic diseases, in EPIC and UKB cohorts 29](#_Toc147753135)

[Table S13: Relative excess risk of all-cancers due to interaction between overweight and/or obesity and incident cardiometabolic diseases, in EPIC and UKB cohorts. Models further adjusted on metformin use 30](#_Toc147753136)

[Table S14: Relative excess risk of all-cancers due to interaction between overweight and/or obesity and incident cardiometabolic diseases, in EPIC and UKB cohorts. Models further adjusted on statins use 31](#_Toc147753137)

# Figure S1: Flowchart with the inclusion and exclusion criteria of the study population, in UK Biobank


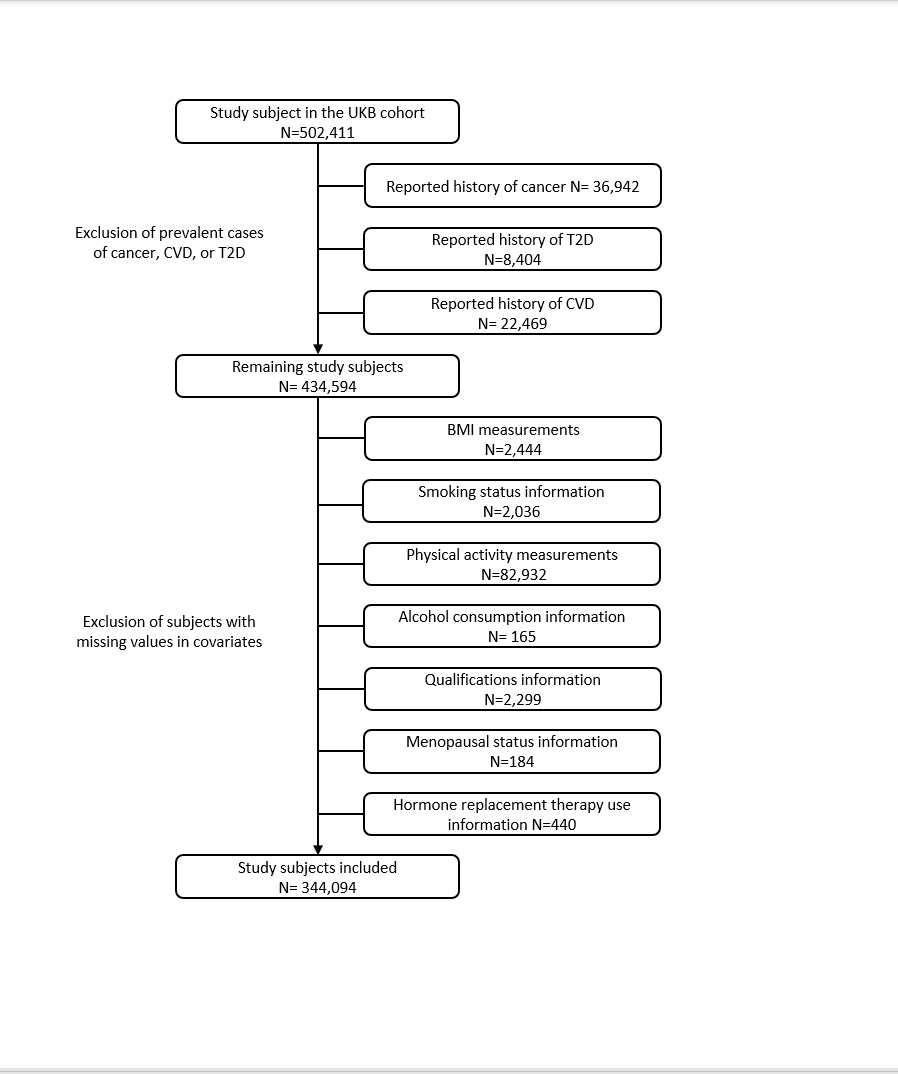


# Figure S2: Flowchart with the inclusion and exclusion criteria of the study population, in EPIC


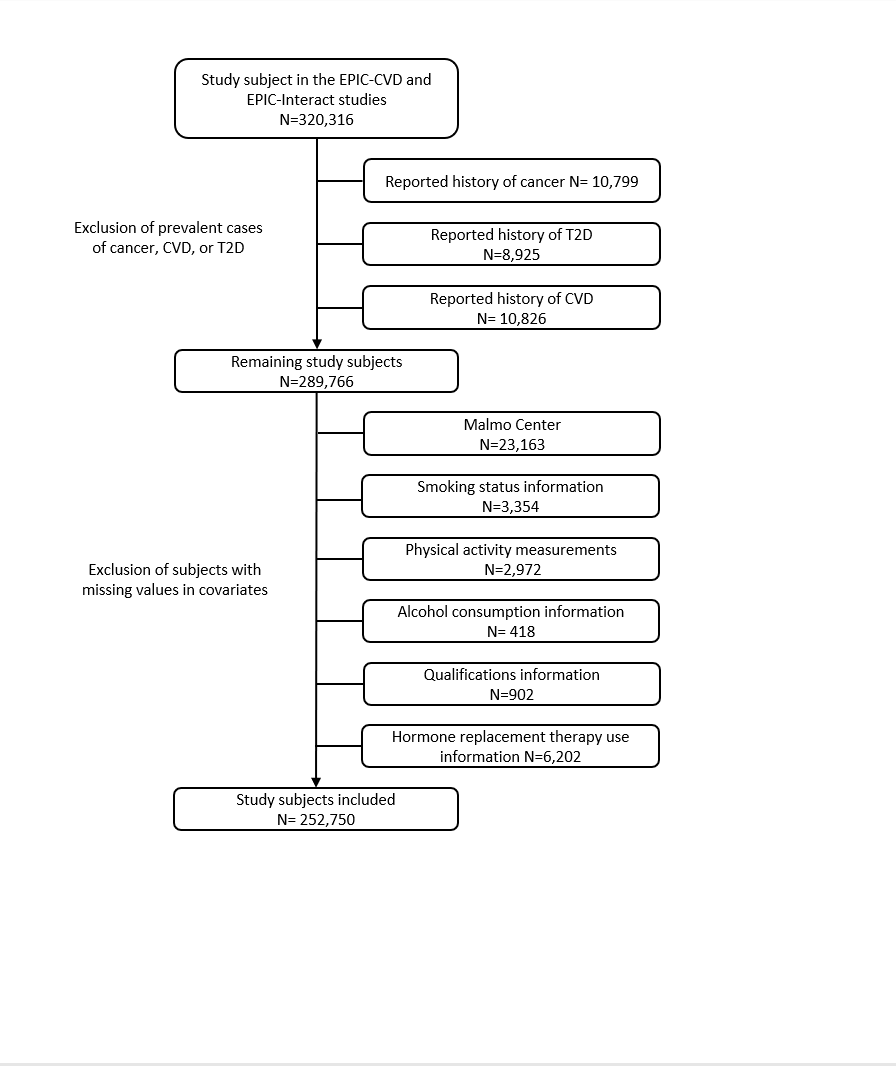


| **Table S1: Diagnostic codes for the definition of cancer, Type 2 Diabetes and cardiovascular diseases** | | | |
| --- | --- | --- | --- |
|  | **ICD10 code** | **ICD9 code** | **ICDO-3 code** |
| Obesity-related cancer | | | |
| Esophagus adenocarcinoma^1^ | C15 | 150 | 8140/3, 8144/3, 8480/3, 8481/3 & 8490/3 |
| Stomach | C16.0 (cardia) | 151 | 8070/3, 8071/3, 8072/3 & 8074/3 |
| Colorectal | C18-C21 | 153-154 |  |
| Liver | C22 | 155 | 8170/3, 8171/3 & 8180/3 (HCC liver) |
| Pancreas | C25 | 157 | 8150/3*, 8151/3*, 8153/3*, 8155/3*, 8240/3*, 8246/3* & 9591/3* |
| Breast | C50 | 174.175 |  |
| Gallbladder - biliary tract | C23-C24 | 156 |  |
| Kidney, except renal pelvis | C64 | 189 | 8312/3 (Kidney) |
| Corpus Uteri | C54-C55 | 179.182 |  |
| ovary | C56 | 183 |  |
| Meningioma | C70 | 192.1, 192.3 | 9530/0 |
| Thyroid | C73 | 193 |  |
| Multiple myeloma | C90 | 203 | 9731 & 9732 |
| Non-obesity related cancer | | | |
| Esophagus SCC | C15 | 150 |  |
| Stomach | C16.1-C16.9 (non-cardia) | 151 |  |
| Larynx | C32 | 161 |  |
| Trachea, bronchus & Lung | C33-C34 | 162 |  |
| Bone and articular cartilage | C40-C41 | 170 |  |
| Malignant melanoma of skin | C43 | 172 |  |
| Connective and soft tissue | C47, C49 | 171 |  |
| Breast premenopausal | C50 | 174, 175 |  |
| Cervix Uteri | C53 | 180 |  |
| Penis | C60 | 187.1-187.4 |  |
| Prostate | C61 | 185 |  |
| Testis | C62 | 186 |  |
| Urinary Tract | C65, C66, C68 | 189.1-189.9 |  |
| Bladder | C67 | 188 |  |
| Hodgkin lymphoma | C81 | 201 | 9670/3, 9671/3, 9673/3, 9675/3, 9678/3, 9679/3, 9680/3, 9684/3, 9687/3, 9689/3, 9690/3, 9691/3, 9695/3, 9698/3, 9699/3, 9700/3, 9700/3, 9700/3, 9700/3, 9701/3, 9702/3, 9705/3, 9709/3, 9714/3, 9717/3, 9718/3, 9719/3, 9727/3, 9728/3, 9731/3, 9732/3, 9733/3, 9734/3, 9761/3, 9820/3, 9823/3, 9826/3, 9827/3, 9831/3, 9832/3, 9833/3, 9835/3, 9836/3, 9837/3, 9940/3 & 9948/3 |
| Non-Hodgkin Lymphoma | C82-C86, C96 | 200, 202 |  |
| Leukemia | C91-C95 | 204-208 | 9800/3, 9801/3, 9802/3, 9820/3, 9821/3, 9824/3, 9825/3, 9830/3, 9835/3, 9836/3, 9840/3, 9841/3, 9850/3, 9860/3, 9861/3, 9863/3, 9866/3, 9867/3, 9868/3, 9872/3, 9873/3, 9874/3, 9875/3, 9876/3, 9891/3, 9895/3, 9900/3, 9910/3, 9931/3, 9932/3 & 9945/3 |
| Brain | C71, C72, C75.1-75.3 | 191,192,194.3,194.4 | 9380/3, 9381/3, 9382/3, 9391/3, 9400/3, 9401/3, 9411/3, 9420/3, 9421/3, 9440/3, 9441/3, 9442/3, 9450/3, 9451/3 & 9473/3 |
| Others and non-specific | C17, C26, C30, C31, C37-C39, C45, C46, C48, C51, C52, C57, C58, C63, C69, C74, C75.0, C75.4-C75.9, C7A, C76, C80, C88, | 152, 158-160, 163-165, 176, 181, 184, 187.5-187.9, 190, 194.0, 194.1, 194.5-194.9, 195, 199, 209.1-209.3, 273.3, 279.5 |  |
| Type 2 Diabetes Mellitus | E11 | - |  |
| Cardiovascular disease |  |  |  |
| Stroke | I60:I69 | 460:434; 436:438 |  |
| Atrial fibrillation | I48 | 427.3 |  |
| Coronary artery disease | I20:I25 | 410:414 |  |

^1^: To determine type of oesophagus, we combine ICD10 code (C15) with ICD-0-3

Abbreviations: ICD-9: International Classification for Diseases, 9^th^; revision; ICD-10: International Classification for Diseases, 10th revision; ICDO-3:3^rd^ Revision of the International Classification of Diseases for Oncology

| Table S2: Main design characteristics of both cohorts | | |
| --- | --- | --- |
|  | **EPIC** [1–3] | **UK Biobank** [4] |
| **Study design**  **Countries**  **Participants**    **Baseline year(s)** | Prospective cohort  Europe, multicentre from 7 countries (Denmark, Germany, Italy, the Netherlands, Spain, Sweden, and the UK)  ~250k men and women aged 35-70 from 7 countries.  1992-2000 | Prospective cohort  The United Kingdom (England, Scotland, Wales)  ~500k men and women from the UK general population aged 40-69  2006-2010 |
| **Follow-up for cancer and cardiometabolic diseases**  Cancer  CVD  T2D | 1992-2013  1992-2010  1992-2007 | 2006-2021  2006-2021  2006-2021 |
| **Definition of cancer and cardiometabolic diseases**  Cancer | - Any first primary cancer^1^ using ICD-10 codes, and in-situ tumor histology.  - Ascertained through cancer registries in Denmark, Italy, the Netherlands, Spain, Sweden, and the United Kingdom, and a combination of health insurance records, cancer pathology registries, and active follow-up in Germany | - Any first primary cancer using ICD-10 codes, and in-situ tumor histology.  - Ascertained with electronic linkage to hospital admissions data |
| CVD | -First coronary heart disease, non-fatal or fatal events among myocardial infarction (I21, I22 ICD-10 codes), other acute or coronary heart disease (I23-I25), haemorrhagic stroke (I60-I61), ischemic stroke (I63), unclassified stroke (I64), or other acute cerebrovascular events (I62, I65-69, F01).  -Ascertained using active follow-up through questionnaires, medical records, hospital morbidity registers, contact with medical professionals, retrieving and assessing death certificates, or verbal autopsy. | -First coronary heart disease, non-fatal or fatal events among myocardial infarction (I21, I22 ICD-10 codes), other acute or coronary heart disease (I23-I25), haemorrhagic stroke (I60-I61), ischemic stroke (I63), unclassified stroke (I64), or other acute cerebrovascular events (I62, I65-69, F01).  -Ascertained with electronic linkage to hospital admissions data. |
| T2D | Diagnosis of type 2 diabetes (ICD-10 code: E11).  -Combination of self-report in the baseline questionnaire, linkage to primary care registers, national diabetes and pharmaceutical registries, secondary-care registers, medication using drug registers, hospital admissions, and mortality data | Diagnosis of type 2 diabetes (ICD-10 code: E11).  -Ascertained with electronic linkage to hospital admissions data. |
| **Incident cases of cancer and cardiometabolic diseases**  Cancer  CVD   T2D | n~8,000  n~10,000   n~11,000 | n~12,000 n~32,000  n~16,000 |
| **Assessment of covariates**  **Age**  **Sex**  **Qualifications**  **Anthropometric measurements**  **Physical Activity**  **Diet***  **Smoking**  **Alcohol**  **Use of hormone therapy**  **Menopausal status (time-varying)** | Age at recruitment  Male/Female  None, primary school compl, technical/professional school, secondary school, longer education (incl. university degree), not specified  Height and weight were measured at recruitment using a standardized protocol, by trained medical staff; in the Oxford center height and weight were self‐reported and then harmonized to reduce heterogeneity  Cambridge physical activity index (inactive, moderately inactive, moderately active, active)  Information from dietary questionnaires. Modified relative Mediterranean Diet Score (mrMDS). An 18-point linear score that incorporates nine nutritional components of the Mediterranean diet: vegetables, legumes, fruits and nuts, cereals, fish and sea food, vegetable oil, and moderate alcohol consumption, and in moderation meat and meat products, and dairy [5].  Smoking status (Never, Former, Current)  Alcohol at recruitment (g/day)  Never, Ever  Premenopausal, Perimenopausal, Postmenopausal, Surgical menopause | Age at recruitment  Male/Female  None of the above, CSEs/O-levels/GCSEs or equivalent, NVQ/HND/HNC/A-levels/AS-levels or equivalent, Other professional quals. (eg nurse/teach), College/university degree, prefer not to answer/missing")  Height and weight were measured at recruitment using a standardized protocol, by trained medical staff  International Physical Activity Questionnaire (IPAQ) activity group (low, medium, high)  UKB Food Frequency questionnaire (FFQ). Healthy diet score was calculated based on consumption of commonly eaten food groups following recommendations on dietary priorities for cardiometabolic health (fruits, vegetables, fish, processed meats, unprocessed red meats, whole grains, refined grains). healthy diet score (range 0 to 6) as described by Hepsomali et al. [6].  Current tobacco smoking (Never, Previous, Current)  Alcohol intake frequency at recruitment (Daily or almost daily, Three or four times a week, Once or twice a week, one to three times a month, Special occasions only, Never, prefer not to answer)  Never, Ever  Premenopausal, Postmenopausal, Unsure because of hysterectomy or other reason |

* The Mediterranean Diet Score (mrMDS) of the EPIC cohort was not available in the UKB cohort, however, in a post-hoc analysis we implemented a healthy diet score in EPIC similar to the one in the UKB and replacing the mrMDS in EPIC by this healthy diet score returned identical risk estimates in our main models.

| Figure S3: Directed Acyclic Graph (DAG) describing the potential causal and confounding effects of obesity on cancer risk |
| --- |
| 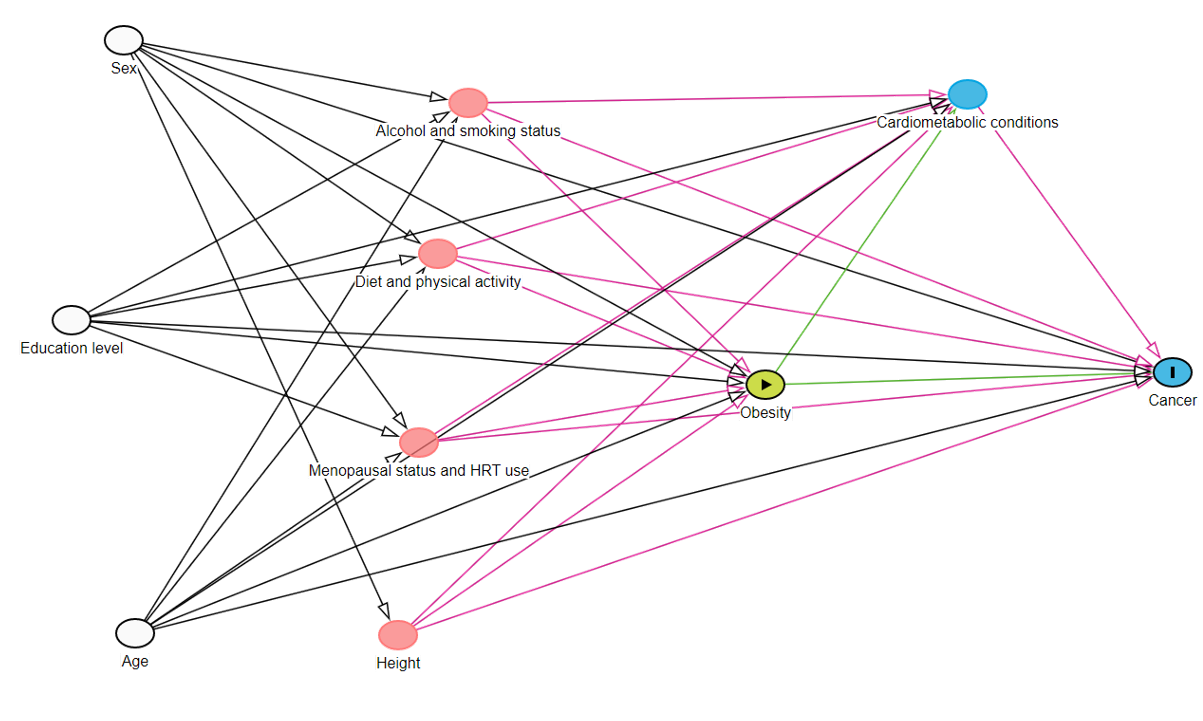 |
| Notes: Green arrow indicate a causal path between the exposure and outcome of interest. Pink arrows indicate biaising path, meaning possible confounding factors modifying the association, between the exposure and the outcome of interest. |

| Figure S4: Framework to study the natural history of multimorbidity in UKB and EPIC with cancer as index disease and T2D and CVD as comorbidities | |
| --- | --- |
| 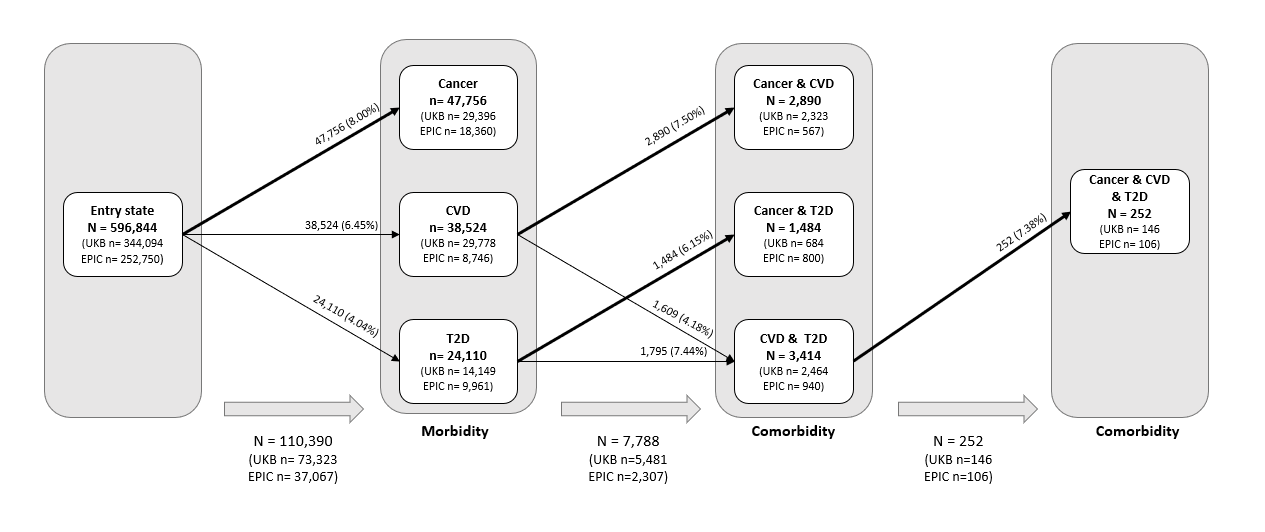 |  |
| Notes: Bold arrows represent the disease-trajectories that were the main interest of this study, cancer is considered as our finale state | |

| **Table S3: Association between BMI (per 1 standard deviation increment^7^) and cancer risk by ascertainment of incident cardiometabolic conditions, in EPIC and UKB cohorts, in men** | | | | | | | | | | | | | | | |
| --- | --- | --- | --- | --- | --- | --- | --- | --- | --- | --- | --- | --- | --- | --- | --- |
|  | **EPIC** | | | | | | | **UKB** | | | | | | | |
|  | N | Cases | HR | 95%CI | p^1^ | p^2^ | p^3^ | N | Cases | HR | 95%CI | p^1^ | p^2^ | p^3^ |  |
| TOTAL CANCERS | | | | | | | | | | | | | | | |
| Overall Unadj.^4^ | 100,297 | 8,308 | 1.02 | (0.99; 1.04) | 0.09 |  |  | 162,866 | 17,523 | 1.01 | (0.99; 1.03) | 0.29 |  |  |  |
| Overall Adj.^5^ | 100,297 | 8,308 | 1.01 | (0.99; 1.04) | 0.18 |  |  | 162,866 | 17,523 | 1.00 | (0.99; 1.02) | 0.47 |  |  |  |
| Baseline^6^ | 100,297 | 7,412 | 1.02 | (0.99; 1.04) | 0.09 |  | 0.37 | 162,866 | 15,386 | 1.00 | (0.99; 1.02) | 0.62 |  | 0.76 |  |
| T2D^6^ | 5,072 | 468 | 0.96 | (0.90; 1.03) | 0.29 | 0.12 |  | 8,626 | 420 | 0.97 | (0.90; 1.07) | 0.54 | 0.48 |  |  |
| CVD^6^ | 5,070 | 343 | 1.02 | (0.93; 1.12) | 0.59 | 0.91 |  | 18,551 | 1,617 | 1.02 | (0.90; 1.08) | 0.33 | 0.44 |  |  |
| T2D-CVD^6^ | 615 | 85 | 0.95 | (0.79; 1.14) | 0.56 | 0.43 |  | 1, 601 | 100 | 1.00 | (0.84; 1.18) | 0.96 | 0.92 |  |  |
| OBESITY-RELATED CANCER | | | | | | | | | | | | | | | |
| Overall Unadj.^4^ | 100,297 | 1,914 | 1.11 | (1.07; 1.16) | <0.001 |  |  | 162,866 | 4,304 | 1.11 | (1.08; 1.15) | <0.001 |  |  |  |
| Overall Adj.^5^ | 100,297 | 1,914 | 1.11 | (1.06; 1.16) | 0.002 |  |  | 162,866 | 4,304 | 1.10 | (1.06; 1.13) | <0.001 |  |  |  |
| Baseline^6^ | 100,297 | 1,712 | 1.11 | (1.06; 1.16) | <0.001 |  | 0.65 | 162,866 | 3,690 | 1.10 | (1.06; 1.14) | <0.001 |  | 0.92 |  |
| T2D^6^ | 5,072 | 124 | 1.11 | (0.98; 1.26) | 0.10 | 0.97 |  | 8,626 | 137 | 1.06 | (0.93; 1.22) | 0.36 | 0.64 |  |  |
| CVD^6^ | 5,070 | 57 | 0.96 | (0.76; 1.20) | 0.73 | 0.21 |  | 18,551 | 437 | 1.10 | (1.00; 1.20) | 0.04 | 0.97 |  |  |
| T2D-CVD^6^ | 615 | 21 | 1.11 | (0.87; 1.60) | 0.58 | 0.98 |  | 1, 601 | 40 | 1.02 | (0.79; 1.32) | 0.85 | 0.58 |  |  |
| NON-OBESITY RELATED CANCERS | | | | | | | | | | | | | | | |
| Overall Unadj.^4^ | 100,297 | 6,394 | 0.99 | (0.97; 1.01) | 0.42 |  |  | 162,866 | 13,269 | 0.98 | (0.96; 0.99) | 0.01 |  |  |  |
| Overall Adj.^5^ | 100,297 | 6,394 | 0.99 | (0.96; 1.01) | 0.31 |  |  | 162,866 | 13,269 | 0.97 | (0.96; 0.99) | 0.005 |  |  |  |
| Baseline^6^ | 100,297 | 5,700 | 0.99 | (0.97; 1.02) | 0.58 |  | 0.13 | 162,866 | 11,746 | 0.97 | (0.95; 0.99) | 0.009 |  | 0.68 |  |
| T2D^6^ | 5,072 | 344 | 0.91 | (0.84; 0.99) | 0.03 | 0.05 |  | 8,626 | 283 | 0.93 | (0.83; 1.02) | 0.15 | 0.36 |  |  |
| CVD^6^ | 5,070 | 286 | 1.03 | (0.94; 1.14) | 0.49 | 0.42 |  | 18,551 | 1,180 | 1.00 | (0.94; 1.05) | 0.90 | 0.46 |  |  |
| T2D-CVD^6^ | 615 | 64 | 0.89 | (0.72; 1.11) | 0.30 | 0.33 |  | 1, 601 | 60 | 0.97 | (0.78; 1.20) | 0.76 | 0.95 |  |  |
| *P-values:*^1^ p-values for association of local association tests; ^2^ p-values for local interaction tests; ^3^ p-value for the global interaction test;  ^4^: for overall non-adjusted model were adjusted on education level, alcohol consumption, smoking status, height, physical activity, healthy diet score and stratified by centre of recruitment and age (5-year categories).  ^5^: for the overall adjusted model (further adjusted on the presence of T2D, CVD and the duration of these comorbidities)  ^6^: models depending on cardiometabolic status (adjusted for the presence of T2D/CVD and for interactions between BMI and presence of T2D/CVD)  ^7:^ Standard deviation (SD) of BMI in EPIC = 3.56; Standard deviation (SD) of BMI in UKB = 4.07  Abbreviations: SD: Standard deviation; BMI: Body mass index; HR: Hazard ratio; 95%CI: 95% Confidence intervals; T2D: Type 2 Diabetes; CVD: Cardiovascular diseases | | | | | | | | | | | | | | | |

| **Figure S5: Forest plot of the associations (with 95% Cis) between BMI and cancer risk depending on cardiometabolic status in EPIC and UKB cohort and the results of the meta-analysis (random effect models), in men** |
| --- |
| 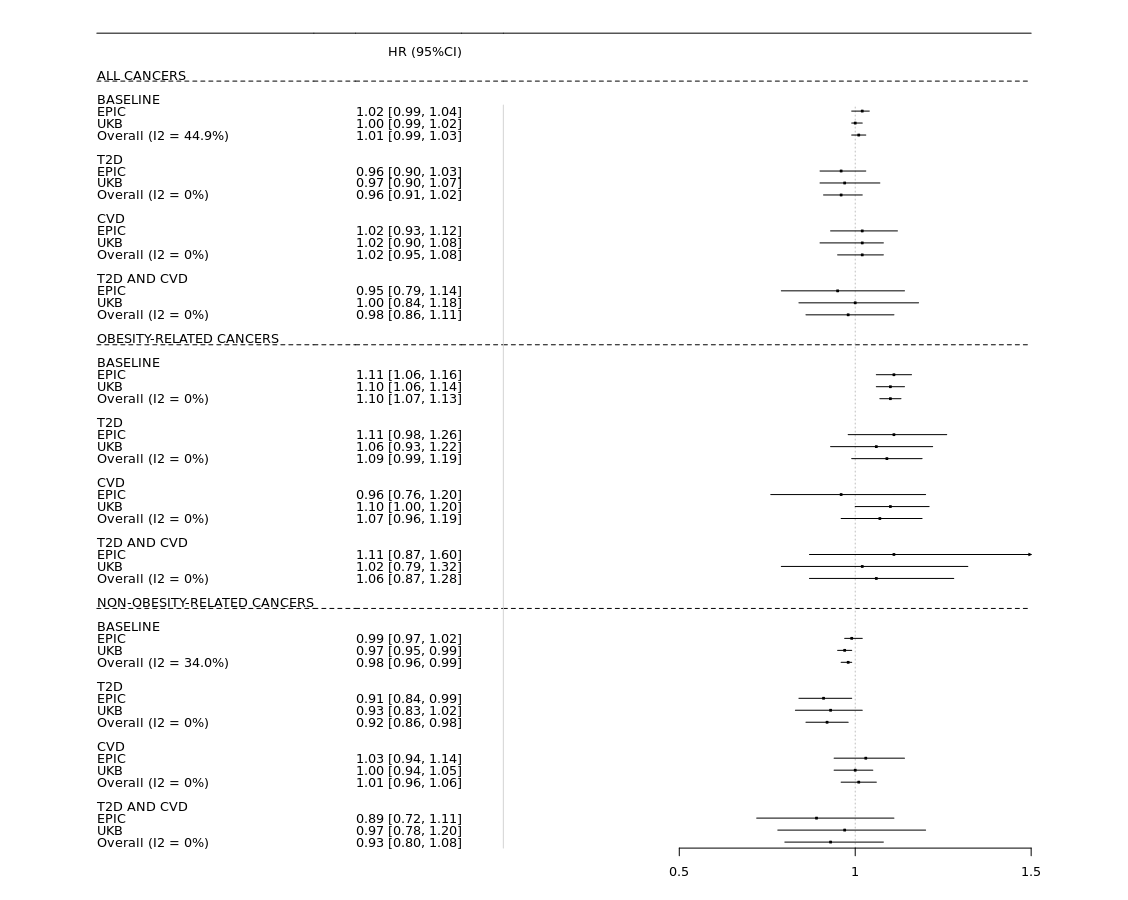 |
| I2 = I² index which quantify the dispersion of effect sizes in a meta-analysis  Abbreviations: SD : Standard deviation ; BMI: Body mass index; HR: Hazard ratio; 95%CI: 95% Confidence intervals; T2D : Type 2 Diabetes ; CVD: Cardiovascular diseases |

| **Table S4: Association between BMI (per 1 standard deviation increment^7^) and cancer risk by ascertainment of incident cardiometabolic conditions, in EPIC and UKB cohorts, in women** | | | | | | | | | | | | | | | |
| --- | --- | --- | --- | --- | --- | --- | --- | --- | --- | --- | --- | --- | --- | --- | --- |
|  | **EPIC** | | | | | | | **UKB** | | | | | | | |
|  | N | Cases | HR | 95%CI | p^1^ | p^2^ | p^3^ | N | Cases | HR | 95%CI | p^1^ | p^2^ | p^3^ |  |
| TOTAL CANCERS | | | | | | | | | | | | | | | |
| Overall Unadj.^4^ | 152,453 | 11,525 | 1.05 | (1.03, 1.07) | <0.001 |  |  | 181,228 | 15,026 | 1.08 | (1.07; 1.10) | <0.001 |  |  |  |
| Overall Adj.^5^ | 152,453 | 11,525 | 1.05 | (1.03, 1.06) | <0.001 |  |  | 181,228 | 15,026 | 1.08 | (1.06; 1.10) | <0.001 |  |  |  |
| Baseline^6^ | 152,453 | 10,948 | 1.04 | (1.03, 1.06) | <0.001 |  | 0.32 | 181,228 | 14,010 | 1.08 | (1.06; 1.10) | <0.001 |  | 0.26 |  |
| T2D^6^ | 4,889 | 332 | 1.05 | (0.99, 1.13) | 0.12 | 0.78 |  | 5,523 | 264 | 1.15 | (1.05; 1.26) | 0.002 | 0.16 |  |  |
| CVD^6^ | 3,676 | 224 | 1.14 | (1.04, 1.25) | 0.007 | 0.08 |  | 11,227 | 706 | 1.13 | (1.06; 1.21) | <0.001 | 0.14 |  |  |
| T2D-CVD^6^ | 325 | 21 | 1.15 | (0.88, 1.51) | 0.31 | 0.48 |  | 863 | 46 | 1.04 | (0.83; 1.30) | 0.71 | 0.77 |  |  |
| OBESITY-RELATED CANCER | | | | | | | | | | | | | | | |
| Overall Unadj.^4^ | 152,453 | 5,978 | 1.10 | (1.07, 1.12) | <0.001 |  |  | 181,228 | 8,272 | 1.17 | (1.15; 1.20) | <0.001 |  |  |  |
| Overall Adj.^5^ | 152,453 | 5,978 | 1.09 | (1.07, 1.12) | <0.001 |  |  | 181,228 | 8,272 | 1.17 | (1.14; 1.19) | <0.001 |  |  |  |
| Baseline^6^ | 152,453 | 5,670 | 1.09 | (1.07, 1.12) | <0.001 |  | 0.58 | 181,228 | 7,714 | 1.16 | (1.14; 1.19) | <0.001 |  | 0.72 |  |
| T2D^6^ | 4,889 | 187 | 1.11 | (1.01, 1.21) | 0.02 | 0.76 |  | 5,523 | 154 | 1.15 | (1.02; 1.30) | 0.02 | 0.88 |  |  |
| CVD^6^ | 3,676 | 111 | 1.19 | (1.04, 1.36) | 0.01 | 0.20 |  | 11,227 | 376 | 1.23 | (1.13; 1.32) | <0.001 | 0.25 |  |  |
| T2D-CVD^6^ | 325 | 10 | 0.97 | (0.64, 1.49) | 0.90 | 0.60 |  | 863 | 28 | 1.15 | (0.88 ;1.52) | 0.31 | 0.94 |  |  |
| NON-OBESITY RELATED CANCERS | | | | | | | | | | | | | | | |
| Overall Unadj.^4^ | 152,453 | 5,547 | 1.00 | (0.98, 1.02) | 0.98 |  |  | 181,228 | 6,654 | 0.99 | (0.96; 1.01) | 0.32 |  |  |  |
| Overall Adj.^5^ | 152,453 | 5,547 | 1.00 | (0.97, 1.02) | 0.83 |  |  | 181,228 | 6,654 | 0.98 | (0.96; 1.01) | 0.21 |  |  |  |
| Baseline^6^ | 152,453 | 5,278 | 1.00 | (0.97, 1.02) | 0.69 |  | 0.29 | 181,228 | 6,296 | 0.98 | (0.95; 1.00) | 0.09 |  | 0.12 |  |
| T2D^6^ | 4,889 | 145 | 0.98 | (0.88, 1.10) | 0.79 | 0.86 |  | 5,523 | 110 | 1.15 | (1.00; 1.31) | 0.04 | 0.02 |  |  |
| CVD^6^ | 3,676 | 113 | 1.09 | (0.95, 1.24) | 0.23 | 0.21 |  | 11,227 | 330 | 1.02 | (0.92; 1.13) | 0.72 | 0.44 |  |  |
| T2D-CVD^6^ | 325 | 11 | 1.32 | (0.91, 1.90) | 0.14 | 0.13 |  | 863 | 18 | 0.87 | (0.60; 1.27) | 0.48 | 0.56 |  |  |
| *P-values:*^1^ p-values for association of local association tests; ^2^ p-values for local interaction tests; ^3^ p-value for the global interaction test;  ^4^: for overall non-adjusted model were adjusted on education level, alcohol consumption, smoking status, height, physical activity, healthy, use of HRT, diet score and stratified by centre of recruitment, menopausal status and age (5-year categories).  ^5^: for the overall adjusted model (further adjusted on the presence of T2D, CVD and the duration of these comorbidities)  ^6^: models depending on cardiometabolic status (adjusted for the presence of T2D/CVD and for interactions between BMI and presence of T2D/CVD)  ^7:^ Standard deviation (SD) of BMI in EPIC = 4.48; Standard deviation (SD) of BMI in UKB = 4.99  Abbreviations: SD: Standard deviation; BMI: Body mass index; HR: Hazard ratio; 95%CI: 95% Confidence intervals; T2D: Type 2 Diabetes; CVD: Cardiovascular diseases | | | | | | | | | | | | | | | |

| **Figure S6: Forest plot of the associations (with 95% CIs) between BMI and cancer risk depending on cardiometabolic status in EPIC and UKB cohort and the results of the meta-analysis (random effect models), in women** |
| --- |
| 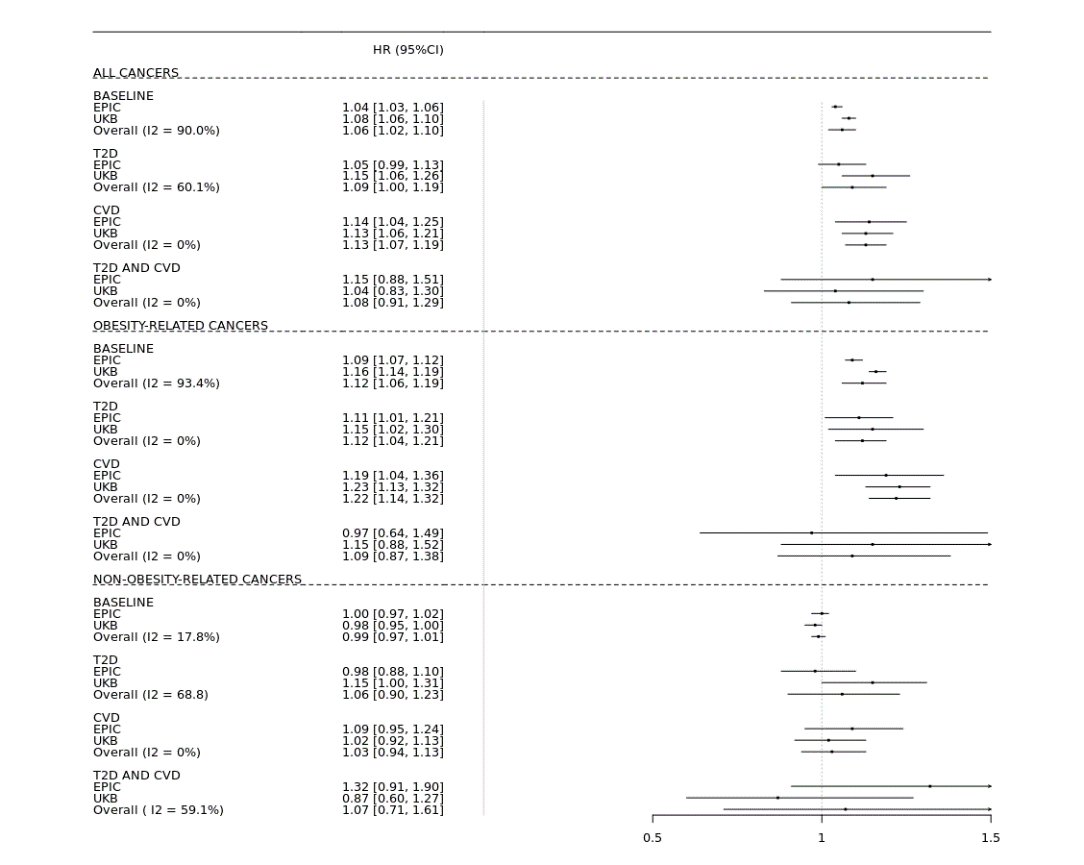 |
| I2 = I² index which quantify the dispersion of effect sizes in a meta-analysis  Abbreviations: SD : Standard deviation ; BMI: Body mass index; HR: Hazard ratio; 95%CI: 95% Confidence intervals; T2D : Type 2 Diabetes ; CVD: Cardiovascular diseases |

| Table S5: Association between BMI (per 1 standard deviation increment^7^) and cancer risk by ascertainment of incident cardiometabolic conditions, with 95% Cis, in UKB. Models further adjusted on metformin use |
| --- |
| \|  \| UKB \| \| \| \| \| \| \| \| \| --- \| --- \| --- \| --- \| --- \| --- \| --- \| --- \| --- \| \| N \| Cases \| HR \| 95%CI \| p^1^ \| p^2^ \| p^3^ \| \| \| **TOTAL CANCER** \| \| \| \| \| \| \| \| \| \| **Overall Unadj.^4^** \| 344,094 \| 32,549 \| 1.05 \| (1.04; 1.06) \| <0.001 \|  \| \|  \| \| **Overall Adj.^5^** \| 344,094 \| 32,549 \| 1.04 \| (1.03; 1.06) \| <0.001 \|  \| \|  \| \| **Baseline^6^** \| 344,094 \| 29,396 \| 1.05 \| (1.03; 1.06) \| <0.001 \|  \| \| 0.54 \| \| **T2D^6^** \| 14,149 \| 684 \| 1.02 \| (0.96; 1.08) \| 0.52 \| 0.34 \| \|  \| \| **CVD^6^** \| 29,778 \| 2,323 \| 1.04 \| (1.00; 1.07) \| 0.04 \| 0.56 \| \|  \| \| **T2D-CVD^6^** \| 2,464 \| 146 \| 0.98 \| (0.87; 1.11) \| 0.80 \| 0.32 \| \|  \| \| **OBESITY-RELATED CANCERS** \| \| \| \| \| \| \| \| \| \| **Overall Unadj.^4^** \| 344,094 \| 12,526 \| 1.16 \| (1.14; 1.19) \| <0.001 \|  \| \|  \| \| **Overall Adj.^5^** \| 344,094 \| 12,526 \| 1.15 \| (1.13; 1.17) \| <0.001 \|  \| \|  \| \| **Baseline^6^** \| 344,094 \| 1,354 \| 1.14 \| (1.12; 1.16) \| <0.001 \|  \| \| 0.58 \| \| **T2D^6^** \| 14,149 \| 291 \| 1.13 \| (1.04; 1.22) \| <0.001 \| 0.73 \| \|  \| \| **CVD^6^** \| 29,778 \| 813 \| 1.19 \| (1.12; 1.25) \| 0.004 \| 0.19 \| \|  \| \| **T2D-CVD^6^** \| 2,464 \| 68 \| 1.12 \| (0.94; 1.32) \| 0.20 \| 0.77 \| \|  \| \| **NON-OBESITY-RELATED CANCERS** \| \| \| \| \| \| \| \| \| \| **Overall Unadj.^4^** \| 344,094 \| 20,023 \| 0.98 \| (0.97; 1.00) \| 0.06 \|  \| \|  \| \| **Overall Adj.^5^** \| 344,094 \| 20,023 \| 0.98 \| (0.97; 0.99) \| 0.03 \|  \| \|  \| \| **Baseline^6^** \| 344,094 \| 18,042 \| 0.99 \| (0.97; 1.00) \| 0.09 \|  \| \| 0.54 \| \| **T2D^6^** \| 14,149 \| 393 \| 0.97 \| (0.89; 1.05) \| 0.42 \| 0.65 \| \|  \| \| **CVD^6^** \| 29,778 \| 1,510 \| 0.96 \| (0.91; 1.00) \| 0.07 \| 0.24 \| \|  \| \| **T2D-CVD^6^** \| 2,464 \| 78 \| 0.92 \| (0.76; 1.10) \| 0.34 \| 0.42 \| \|  \| |
| *Pvalues:*^1^ p-values for association of local association tests; ^2^ p-values for local interaction tests; ^3^ p-value for the global interaction test;  ^4^: for overall non-adjusted model were adjusted on education level, alcohol consumption, smoking status, height, physical activity, healthy diet score, menopausal status, use of HRT, metformin use and stratified by centre of recruitment, age (5-year categories) and sex.  ^5^: for the overall adjusted model (further adjusted on the presence of T2D, CVD and the duration of these comorbidities)  ^6^: models depending on cardiometabolic status (adjusted for the presence of T2D/CVD and for interactions between BMI and presence of T2D/CVD)  ^7:^ Standard deviation (SD) of BMI in UKB = 4.60  Abbreviations: SD: Standard deviation; BMI: Body mass index; HR: Hazard ratio; 95%CI: 95% Confidence intervals; T2D: Type 2 Diabetes; CVD: Cardiovascular diseases |

| Table S6: Association between body mass index (per one standard deviation increment^7^) and cancer risk by ascertainment of incident cardiometabolic conditions, with 95% CIs, in UKB. Models further adjusted on statins use | | | | | | | | |
| --- | --- | --- | --- | --- | --- | --- | --- | --- |
|  | **UKB –** | | | | | | | |
|  | N | Cases | HR | 95%CI | p^1^ | p^2^ | p^3^ | |
| **TOTAL CANCER** | | | | | | | | |
| **Overall Unadj.^4^** | 344,094 | 32,549 | 1.05 | (1.03; 1.06) | <0.001 |  | |  |
| **Overall Adj.^5^** | 344,094 | 32,549 | 1.04 | (1.03; 1.06) | <0.001 |  | |  |
| **Baseline^6^** | 344,094 | 29,396 | 1.05 | (1.03; 1.06) | <0.001 |  | | 0.44 |
| **T2D^6^** | 14,149 | 684 | 1.01 | (0.95; 1.07) | 0.68 | 0.25 | |  |
| **CVD^6^** | 29,778 | 2,323 | 1.04 | (1.00; 1.08) | 0.03 | 0.66 | |  |
| **T2D-CVD^6^** | 2,464 | 146 | 0.97 | (0.86; 1.10) | 0.70 | 0.27 | |  |
| **OBESITY-RELATED CANCERS** | | | | | | | | |
| **Overall Unadj.^4^** | 344,094 | 12,526 | 1.15 | (1.13; 1.17) | <0.001 |  | |  |
| **Overall Adj.^5^** | 344,094 | 12,526 | 1.15 | (1.12; 1.17) | <0.001 |  | |  |
| **Baseline^6^** | 344,094 | 1,354 | 1.14 | (1.12; 1.16) | <0.001 |  | | 0.52 |
| **T2D^6^** | 14,149 | 291 | 1.12 | (1.03; 1.22) | <0.001 | 0.66 | |  |
| **CVD^6^** | 29,778 | 813 | 1.19 | (1.13; 1.25) | <0.001 | 0.17 | |  |
| **T2D-CVD^6^** | 2,464 | 68 | 1.11 | (0.94; 1.31) | 0.22 | 0.73 | |  |
| **NON-OBESITY-RELATED CANCERS** | | | | | | | | |
| **Overall Unadj.^4^** | 344,094 | 20,023 | 0.98 | (0.97; 0.99) | 0.04 |  | |  |
| **Overall Adj.^5^** | 344,094 | 20,023 | 0.98 | (0.97; 0.99) | 0.02 |  | |  |
| **Baseline^6^** | 344,094 | 18,042 | 0.98 | (0.97; 1.00) | 0.07 |  | | 0.53 |
| **T2D^6^** | 14,149 | 393 | 0.96 | (0.89; 1.04) | 0.32 | 0.53 | |  |
| **CVD^6^** | 29,778 | 1,510 | 0.96 | (0.92; 1.01) | 0.09 | 0.30 | |  |
| **T2D-CVD^6^** | 2,464 | 78 | 0.91 | (0.76; 1.09) | 0.29 | 0.37 | |  |
| *Pvalues:*^1^ p-values for association of local association tests; ^2^ p-values for local interaction tests; ^3^ p-value for the global interaction test;  ^4^: for overall non-adjusted model were adjusted on education level, alcohol consumption, smoking status, height, physical activity, healthy diet score, menopausal status, use of HRT, statin use and stratified by centre of recruitment, age (5-year categories) and sex.  ^5^: for the overall adjusted model (further adjusted on the presence of T2D, CVD and the duration of these comorbidities)  ^6^: models depending on cardiometabolic status (adjusted for the presence of T2D/CVD and for interactions between BMI and presence of T2D/CVD)  ^7:^ Standard deviation (SD) of BMI in UKB = 4.60  Abbreviations: SD: Standard deviation; BMI: Body mass index; HR: Hazard ratio; 95%CI: 95% Confidence intervals; T2D: Type 2 Diabetes; CVD: Cardiovascular diseases | | | | | | | | |

| **Table S7: Association between BMI (per 1 standard deviation increment^7^) and cancer risk by ascertainment of incident cardiometabolic conditions, in EPIC and UKB cohorts, among never smokers** | | | | | | | | | | | | | | | |
| --- | --- | --- | --- | --- | --- | --- | --- | --- | --- | --- | --- | --- | --- | --- | --- |
|  | **EPIC** | | | | | | | **UKB** | | | | | | | |
|  | N | Cases | HR | 95%CI | p^1^ | p^2^ | p^3^ | N | Cases | HR | 95%CI | p^1^ | p^2^ | p^3^ |  |
| TOTAL CANCERS | | | | | | | | | | | | | | | |
| Overall Unadj.^4^ | 116,618 | 7,799 | 1.07 | (1.05; 1.09) | <0.001 |  |  | 192,956 | 16,106 | 1.07 | (1.06; 1.09) | <0.001 |  |  |  |
| Overall Adj.^5^ | 116,618 | 7,799 | 1.06 | (1.04; 1.08) | <0.001 |  |  | 192,956 | 16,106 | 1.07 | (1.05; 1.09) | <0.001 |  |  |  |
| Baseline^6^ | 116,618 | 7,367 | 1.07 | (1.05; 1.09) | <0.001 |  | 0.49 | 192,956 | 14,829 | 1.07 | (1.05; 1.09) | <0.001 |  | 0.23 |  |
| T2D^6^ | 3,935 | 259 | 1.00 | (0.92; 1.09) | 0.92 | 0.16 |  | 6,512 | 274 | 0.98 | (0.90; 1.07) | 0.69 | 0.06 |  |  |
| CVD^6^ | 2,725 | 153 | 1.02 | (0.90; 1.16) | 0.71 | 0.52 |  | 14,188 | 947 | 1.09 | (1.03; 1.15) | 0.001 | 0.45 |  |  |
| T2D-CVD^6^ | 264 | 20 | 1.02 | (0.76; 1.38) | 0.87 | 0.79 |  | 999 | 56 | 1.05 | (0.86; 1.27) | 0.65 | 0.82 |  |  |
| OBESITY-RELATED CANCER | | | | | | | | | | | | | | | |
| Overall Unadj.^4^ | 116,618 | 3,680 | 1.12 | (1.09; 1.15) | <0.001 |  |  | 192,956 | 6,543 | 1.18 | (1.16; 1.21) | <0.001 |  |  |  |
| Overall Adj.^5^ | 116,618 | 3,680 | 1.12 | (1.09; 1.15) | <0.001 |  |  | 192,956 | 6,543 | 1.17 | (1.14; 1.20) | <0.001 |  |  |  |
| Baseline^6^ | 116,618 | 3,478 | 1.12 | (1.09; 1.15) | <0.001 |  | 0.80 | 192,956 | 6,013 | 1.17 | (1.14; 1.20) | <0.001 |  | 0.49 |  |
| T2D^6^ | 3,935 | 125 | 1.07 | (0.95; 1.20) | 0.26 | 0.43 |  | 6,512 | 130 | 1.10 | (0.97; 1.24) | 0.14 | 0.31 |  |  |
| CVD^6^ | 2,725 | 71 | 1.17 | (0.98; 1.39) | 0.07 | 0.61 |  | 14,188 | 372 | 1.22 | (1.13; 1.32) | <0.001 | 0.26 |  |  |
| T2D-CVD^6^ | 264 | 6 | 1.01 | (0.52; 1.94) | 0.97 | 0.76 |  | 999 | 28 | 1.16 | (0.89; 1.52) | 0.27 | 0.96 |  |  |
| NON-OBESITY RELATED CANCERS | | | | | | | | | | | | | | | |
| Overall Unadj.^4^ | 116,618 | 4,119 | 1.02 | (0.99; 1.04) | 0.27 |  |  | 192,956 | 9,563 | 1.00 | (0.98; 1.02) | 0.93 |  |  |  |
| Overall Adj.^5^ | 116,618 | 4,119 | 1.01 | (0.98; 1.04) | 0.34 |  |  | 192,956 | 9,563 | 1.00 | (0.98; 1.02) | 0.80 |  |  |  |
| Baseline^6^ | 116,618 | 3,889 | 1.02 | (0.99; 1.04) | 0.18 |  | 0.32 | 192,956 | 8,816 | 1.00 | (0.97; 1.02) | 0.84 |  | 0.59 |  |
| T2D^6^ | 3,935 | 134 | 0.94 | (0.83; 1.06) | 0.32 | 0.20 |  | 6,512 | 144 | 0.92 | (0.81; 1.05) | 0.21 | 0.23 |  |  |
| CVD^6^ | 2,725 | 82 | 0.90 | (0.75; 1.08) | 0.25 | 0.17 |  | 14,188 | 575 | 1.02 | (0.95; 1.10) | 0.59 | 0.56 |  |  |
| T2D-CVD^6^ | 264 | 14 | 1.02 | (0.72; 1.43) | 0.93 | 0.98 |  | 999 | 28 | 1.01 | (0.76; 1.35) | 0.93 | 0.92 |  |  |
| *P-values:*^1^ p-values for association of local association tests; ^2^ p-values for local interaction tests; ^3^ p-value for the global interaction test;  ^4^: for overall non-adjusted model were adjusted on education level, alcohol consumption, height, physical activity, healthy diet score, menopausal status, use of HRT and stratified by centre of recruitment, sex and age (5-year categories).  ^5^: for the overall adjusted model (further adjusted on the presence of T2D, CVD and the duration of these comorbidities)  ^6^: models depending on cardiometabolic status (adjusted for the presence of T2D/CVD and for interactions between BMI and presence of T2D/CVD)  ^7:^ Standard deviation (SD) of BMI in EPIC = 4.36; Standard deviation (SD) of BMI in UKB = 4.61  Abbreviations: SD: Standard deviation; BMI: Body mass index; HR: Hazard ratio; 95%CI: 95% Confidence intervals; T2D: Type 2 Diabetes; CVD: Cardiovascular diseases | | | | | | | | | | | | | | | |

| **Table S8: Association between BMI (per 1 standard deviation increment^7^) and cancer risk (total cancers and non-obesity-related cancers) by ascertainment of incident cardiometabolic conditions, in EPIC and UKB cohorts** | | | | | | | | | | | | | | |
| --- | --- | --- | --- | --- | --- | --- | --- | --- | --- | --- | --- | --- | --- | --- |
|  | **EPIC** | | | | | | | **UKB** | | | | | | |
|  | N | Cases | HR | 95%CI | p^1^ | p^2^ | p^3^ | N | Cases | HR | 95%CI | p^1^ | p^2^ | p^3^ |
| TOTAL CANCERS | | | | | | | | | | | | | | |
| Overall Unadj.^4^ | 252,750 | 19,833 | 1.04 | (1.02; 1.05) | <0.001 |  |  | 344,094 | 32,549 | 1.05 | (1.04; 1.06) | <0.001 |  |  |
| Overall Adj.^5^ | 252,750 | 19,833 | 1.03 | (1.02; 1.05) | <0.001 |  |  | 344,094 | 32,549 | 1.04 | (1.03; 1.05) | <0.001 |  |  |
| Baseline^6^ | 252,750 | 18,360 | 1.03 | (1.02; 1.05) | <0.001 |  | 0.54 | 344,094 | 29,396 | 1.04 | (1.03; 1.06) | <0.001 |  | 0.48 |
| T2D^6^ | 9,961 | 800 | 1.01 | (0.96; 1.06) | 0.66 | 0.36 |  | 14,149 | 684 | 1.01 | (0.95; 1.07) | 0.71 | 0.27 |  |
| CVD^6^ | 8,746 | 567 | 1.07 | (1.00; 1.14) | 0.04 | 0.31 |  | 29,778 | 2,323 | 1.04 | (1.00; 1.07) | 0.04 | 0.67 |  |
| T2D-CVD^6^ | 940 | 106 | 0.99 | (0.86; 1.16) | 0.97 | 0.64 |  | 2,464 | 146 | 0.97 | (0.86; 1.10) | 0.69 | 0.28 |  |
| NON-OBESITY-RELATED CANCERS | | | | | | | | | | | | | | |
| Overall Unadj.^4^ | 252,750 | 11,941 | 0.99 | (0.98; 1.01) | 0.46 |  |  | 344,094 | 20,023 | 0.98 | (0.97; 0.99) | 0.02 |  |  |
| Overall Adj.^5^ | 252,750 | 11,941 | 0.99 | (0.97; 1.01) | 0.31 |  |  | 344,094 | 20,023 | 0.98 | (0.96; 0.99) | 0.005 |  |  |
| Baseline^6^ | 252,750 | 10,978 | 0.99 | (0.97; 1.01) | 0.41 |  | 0.23 | 344,094 | 18,042 | 0.98 | (0.97; 0.99) | 0.02 |  | 0.56 |
| T2D^6^ | 9,961 | 489 | 0.94 | (0.88; 1.00) | 0.07 | 0.11 |  | 14,149 | 393 | 0.96 | (0.88; 1.04) | 0.30 | 0.56 |  |
| CVD^6^ | 8,746 | 399 | 1.05 | (0.96; 1.13) | 0.27 | 0.20 |  | 29,778 | 1,510 | 0.96 | (0.91; 1.00) | 0.07 | 0.30 |  |
| T2D-CVD^6^ | 940 | 75 | 0.97 | (0.81; 1.17) | 0.78 | 0.84 |  | 2,464 | 78 | 0.91 | (0.75; 1.09) | 0.28 | 0.38 |  |
| *P-values:*^1^ p-values for association of local association tests; ^2^ p-values for local interaction tests; ^3^ p-value for the global interaction test;  ^4^: for overall non-adjusted model were adjusted on education level, alcohol consumption, smoking status, height, physical activity, healthy diet score, menopausal status, use of HRT and stratified by centre of recruitment, age (5-year categories) and sex.  ^5^: for the overall adjusted model (further adjusted on the presence of T2D, CVD and the duration of these comorbidities)  ^6^: models depending on cardiometabolic status (adjusted for the presence of T2D/CVD and for interactions between BMI and presence of T2D/CVD)  ^7:^ Standard deviation (SD) of BMI in EPIC = 4.18; Standard deviation (SD) of BMI in UKB = 4.60  Abbreviations: SD: Standard deviation; BMI: Body mass index; HR: Hazard ratio; 95%CI: 95% Confidence intervals; T2D: Type 2 Diabetes; CVD: Cardiovascular diseases | | | | | | | | | | | | | | |

| **Figure S7: Forest plot of the associations (with 95% CIs) between BMI and cancer risk (total cancers and non-obesity-related cancers) by ascertainment of incident cardiometabolic conditions, in EPIC and UKB cohort and the results of the meta-analysis (random effect models)** |
| --- |
| 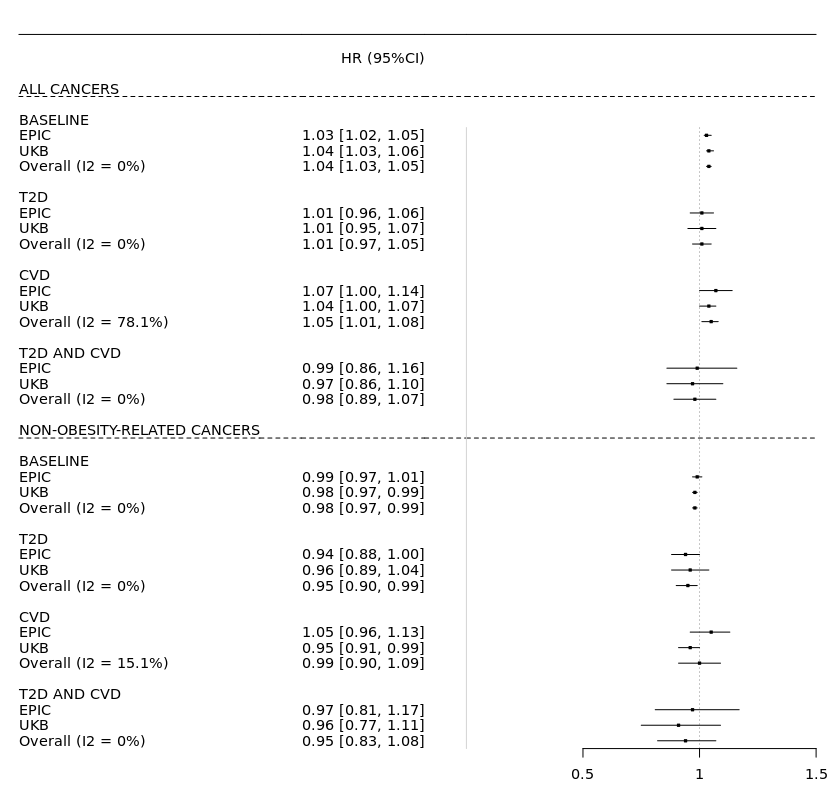 |
| I2 = I² index which quantify the dispersion of effect sizes in a meta-analysis  Abbreviations: HR: Hazard ratio; 95%CI: 95% Confidence intervals; T2D : Type 2 Diabetes ; CVD: Cardiovascular diseases |

| **Table S9: Relative excess risk of all-cancers due to interaction between overweight and/or obesity and incident cardiometabolic diseases, in EPIC and UKB cohorts, in men.** |
| --- |
| \|  \| **EPIC** \| \| \| \| \| **UKB** \| \| \| \| --- \| --- \| --- \| --- \| --- \| --- \| --- \| --- \| --- \| \| **T2D** \| \| **CVD** \| \| **T2D and CVD** \| **T2D** \| **CVD** \| **T2D and CVD** \| \| **ALL CANCERS** \| \| \| \| \| \| \| \| \| \| **BMI<25 kg/m^2^, without disease^1^ of interest** \| 1.00 (ref) \| 1.00 (ref) \| \| 1.00 (ref) \| \| 1.00 (ref) \| 1.00 (ref) \| 1.00 (ref) \| \| **BMI<25 kg/m^2^, with disease** \| 1.29 (0.90; 1.85) \| 1.44 (1.07; 1.95) \| \| 2.19 (1.06; 4.54) \| \| 1.68 (1.16; 2.44) \| 2.20 (1.90; 2.53) \| 2.72 (1.10; 6.73) \| \| **BMI≥25 kg/m^2^, without disease^1^ of interest** \| 0.95 (0.91; 1.00) \| 0.95 (0.91; 1.00) \| \| 0.95 (0.91; 1.00) \| \| 1.04 (1.00; 1.08) \| 1.04 (1.00; 1.08) \| 1.04 (1.00; 1.08) \| \| **BMI≥25 kg/m^2^, with disease (joint effect)** \| 1.20 (0.92; 1.55) \| 1.37 (1.06; 1.76) \| \| 1.61 (1.03; 2.49) \| \| 1.83 (1.48; 2.27) \| 2.38 (2.13; 2.66) \| 2.93 (2.90; 5.32) \| \| **RERI (95%CI)** \| **-0.05 (-0.42; 0.32)** \| **-0.03 (-0.28; 0.23)** \| \| **-0.54 (-2.00; 0.92)** \| \| **0.10 (-0.46; 0.67)** \| **0.14 (-0.13; 0.41)** \| **1.16 (-1.38; 3.70)** \| \| **BMI<30 kg/m^2^, without disease^1^ of interest** \| 1.00 (ref) \| \| 1.00 (ref) \| \| 1.00 (ref) \| 1.00 (ref) \| 1.00 (ref) \| 1.00 (ref) \| \| **BMI<30 kg/m^2^, with disease** \| 1.75 (1.43; 2.13) \| \| 1.38 (1.12; 1.71) \| \| 2.30 (1.61; 3.28) \| 1.80 (1.43; 2.26) \| 2.24 (2.01; 2.50) \| 4.27 (3.00; 6.09) \| \| **BMI≥30 kg/m^2^, without disease^1^ of interest** \| 1.09 (1.03; 1.15) \| \| 1.06 (1.00; 1.12) \| \| 1.07 (1.01; 1.13) \| 0.99 (0.96; 1.03) \| 0.99 (0.95; 1.02) \| 0.99 (0.96; 1.03) \| \| **BMI≥30 kg/m^2^, with disease (joint effect)** \| 1.58 (1.27; 1.96) \| \| 1.58 (1.18; 2.12) \| \| 2.02 (1.33; 3.06) \| 1.71 (1.36; 2.16) \| 2.32 (2.03; 2.64) \| 3.26 (2.28; 4.66) \| \| **RERI (95%CI)** \| **-0.26 (-0.52; 0.01)** \| \| **0.14 (-0.24; 0.52)** \| \| **-0.35 (-1.15; 0.73)** \| **-0.07 (-0.41; 0.26)** \| **0.09 (-0.17; 0.34)** \| **-1.01 (-2.52; 0.50)** \| \| **OBESITY-RELATED CANCERS** \| \| \| \| \| \| \| \| \| \| **BMI<25 kg/m^2^, without disease^1^ of interest** \| 1.00 (ref) \| \| 1.00 (ref) \| \| 1.00 (ref) \| 1.00 (ref) \| 1.00 (ref) \| 1.00 (ref) \| \| **BMI<25 kg/m^2^, with disease** \| 1.67 (0.81; 3.44) \| \| 1.14 (0.54; 2.37) \| \| 2.11 (0.26; 19.51) \| 2.19 (1.09; 4.43) \| 3.29 (2.51; 4.30) \| 6.38 (1.51; 26.98) \| \| **BMI≥25 kg/m^2^, without disease^1^ of interest** \| 1.05 (0.95; 1.17) \| \| 1.06 (0.96; 1.18) \| \| 1.06 (0.99; 1.19) \| 1.21 (1.12; 1.30) \| 1.21 (1.12; 1.31) \| 1.21 (1.12 1.30) \| \| **BMI≥25 kg/m^2^, with disease (joint effect)** \| 2.25 (1.44; 3.51) \| \| 1.04 (0.55; 1.94) \| \| 2.91 (2.37; 9.57) \| 2.91 (2.01; 4.21) \| 3.89 (3.20; 4.74) \| 9.16 (5.54; 15.14) \| \| **RERI (95%CI)** \| **0.52 (-0.59; 1.65)** \| \| **-0.16 (-0.81; 0.49)** \| \| **0.74 (-7.25; 5.78)** \| **0.51 (-0.96; 1.98)** \| **0.39 (-0.43; 1.21)** \| **2.57 (-6.87; 12.01)** \| \| **BMI<30 kg/m^2^, without disease^1^ of interest** \| 1.00 (ref) \| \| 1.00 (ref) \| \| 1.00 (ref) \| 1.00 (ref) \| 1.00 (ref) \| 1.00 (ref) \| \| **BMI<30 kg/m^2^, with disease** \| 3.42 (2.45; 4.77) \| \| 1.45 (0.89; 2.38) \| \| 3.62 (1.68; 7.83) \| 2.60 (1.74; 3.88) \| 3.25 (2.68; 3.94) \| 9.53 (5.34; 17.02) \| \| **BMI≥30 kg/m^2^, without disease^1^ of interest** \| 1.36 (1.21; 1.52) \| \| 1.32 (1.18; 1.47) \| \| 1.29 (1.15; 1.43) \| 1.19 (1.11; 1.28) \| 1.19 (1.10; 1.28) \| 1.19 (1.11; 1.28) \| \| **BMI≥30 kg/m^2^, with disease (joint effect)** \| 3.22 (2.22; 4.68) \| \| 0.80 (0.34; 1.89) \| \| 6.67 (3.09; 14.41) \| 2.55 (1.71; 3.81) \| 3.74 (2.96; 4.73) \| 6.97 (3.88; 12.53) \| \| **RERI (95%CI)** \| **-0.55 (-1.54; 0.43)** \| \| **-0.97 (-1.73; -0.21)** \| \| **2.76 (-1.51; 7.03)** \| **-0.24 (-1.11; 0.63)** \| **0.31 (-0.44; 1.06)** \| **-2.75 (-8.02; 2.53)** \| |
| Notes: The model included BMI as a binary variable with an interaction term with the binary variable of the existence of the disease of interest and was adjusted by duration of comorbidities, education levels, smoking status, alcohol consumption, height, physical activity, healthy diet score, two binary variables of the two cardiometabolic conditions status not studied and stratified by, centre and age at recruitment (5-year categories)  RERI_RR_ = RR_11_ − RR_10_ − RR_01_ + 1, where _11_ denotes being exposed to both factors (eg, overweight/obesity and T2D), _10_ to one factor (eg, overweight/obesity), and _01_ to the other one (eg, *T2D*). A RERI of 0 was considered a lack of additive interaction and 95%CIs were calculated as proposed by *Hosmer and Lemeshow*.  Abbreviations: BMI: Body mass index; T2D: Type 2 Diabetes; CVD: Cardiovascular diseases; RERI: Relative excessive Risk due to Interaction  ^1^ without diseases means without the combination of the disease(s) studied. |

| Figure S8: Forest plot of the relative excess risk of all-cancers due to interaction (with 95% CIs) between overweight and/or obesity and incident cardiometabolic diseases in EPIC and UKB cohort and the results of the meta-analysis (random effect models), in men |
| --- |
| 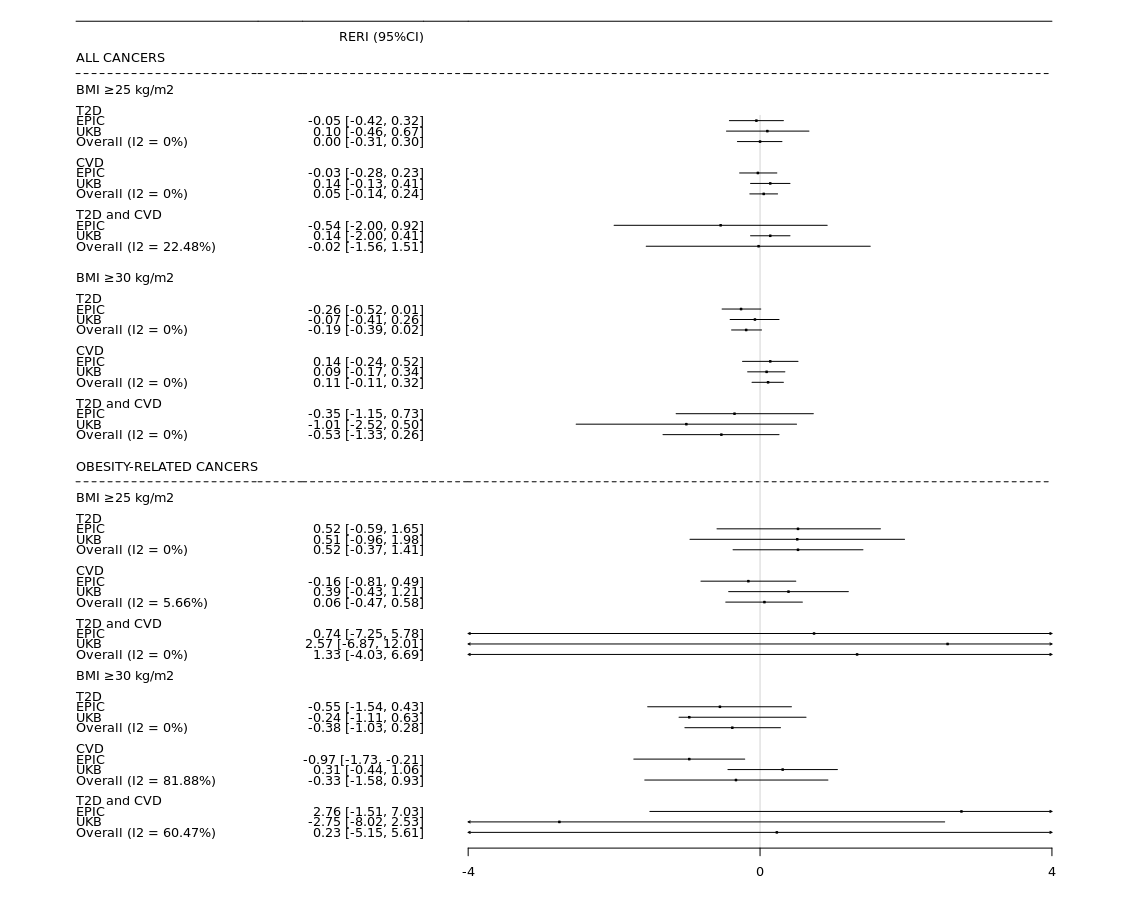 |
| I2 = I² index which quantify the dispersion of effect sizes in a meta-analysis  Abbreviations: SD : Standard deviation ; BMI: Body mass index; RERI: Relative excess risk due to interaction; 95%CI: 95% Confidence intervals; T2D : Type 2 Diabetes ; CVD: Cardiovascular diseases |

| **Table S10: Relative excess risk of all-cancers due to interaction between overweight and/or obesity and incident cardiometabolic diseases, in EPIC and UKB cohorts, in women.** |
| --- |
| \|  \| **EPIC** \| \| \| \| \| **UKB** \| \| \| \| --- \| --- \| --- \| --- \| --- \| --- \| --- \| --- \| --- \| \| **T2D** \| \| **CVD** \| \| **T2D and CVD** \| **T2D** \| **CVD** \| **T2D and CVD** \| \| **ALL CANCERS** \| \| \| \| \| \| \| \| \| \| **BMI<25 kg/m^2^, without disease^1^ of interest** \| 1.00 (ref) \| 1.00 (ref) \| \| 1.00 (ref) \| \| 1.00 (ref) \| 1.00 (ref) \| 1.00 (ref) \| \| **BMI<25 kg/m^2^, with disease** \| 1.72 (1.26; 2.35) \| 1.93 (1.45; 2.58) \| \| 2.39 (0.97; 5.90) \| \| 1.45 (0.87; 2.42) \| 2.39 (1.97; 2.90) \| 3.83 (1.37; 10.73) \| \| **BMI≥25 kg/m^2^, without disease^1^ of interest** \| 1.04 (1.00; 1.08) \| 1.04 (1.00; 1.08) \| \| 1.04 (1.00; 1.08) \| \| 1.11 (1.07; 1.15) \| 1.11 (1.07; 1.15) \| 1.11 (1.07; 1.15) \| \| **BMI≥25 kg/m^2^, with disease (joint effect)** \| 2.12 (1.70; 2.66) \| 2.21 (1.68; 2.89) \| \| 3.26 (2.01; 5.29) \| \| 2.09 (1.56; 2.80) \| 2.82 (2.40; 3.32) \| 4.48 (2.89; 6.95) \| \| **RERI (95%CI)** \| **0.36 (-0.12; 0.84)** \| **0.23 (-0.27; 0.74)** \| \| **0.83 (-1.45; 3.11)** \| \| **0.53 (-0.18; 1.24)** \| **0.33 (-0.09; 0.74)** \| **0.54 (-3.47; 4.55)** \| \| **BMI<30 kg/m^2^, without disease^1^ of interest** \| 1.00 (ref) \| \| 1.00 (ref) \| \| 1.00 (ref) \| 1.00 (ref) \| 1.00 (ref) \| 1.00 (ref) \| \| **BMI<30 kg/m^2^, with disease** \| 2.00 (1.58; 2.53) \| \| 1.89 (1.46; 2.44) \| \| 2.96 (1.71; 5.10) \| 1.62 (1.16; 2.26) \| 2.37 (2.01; 2.78) \| 3.95 (2.21; 7.06) \| \| **BMI≥30 kg/m^2^, without disease^1^ of interest** \| 1.08 (1.03; 1.13) \| \| 1.06 (1.01; 1.12) \| \| 1.08 (1.03; 1.13) \| 1.13 (1.09; 1.18) \| 1.12 (1.08; 1.17) \| 1.13 (1.09; 1.18) \| \| **BMI≥30 kg/m^2^, with disease (joint effect)** \| 2.08 (1.63; 2.65) \| \| 2.95 (2.12; 4.11) \| \| 3.24 (1.82; 5.77) \| 2.20 (1.62; 2.98) \| 3.16 (2.63; 3.81) \| 4.46 (2.76; 7.21) \| \| **RERI (95%CI)** \| **0.00 (-0.19; 0.19)** \| \| **1.00 (0.18; 1.81)** \| \| **0.21 (-1.71; 2.13)** \| **0.45 (-0.05; 0.94)** \| **0.67 (0.20; 1.14)** \| **0.37 (-2.16; 2.90)** \| \| **OBESITY-RELATED CANCERS** \| \| \| \| \| \| \| \| \| \| **BMI<25 kg/m^2^, without disease^1^ of interest** \| 1.00 (ref) \| \| 1.00 (ref) \| \| 1.00 (ref) \| 1.00 (ref) \| 1.00 (ref) \| 1.00 (ref) \| \| **BMI<25 kg/m^2^, with disease** \| 1.88 (1.13; 3.12) \| \| 1.71 (1.06; 2.78) \| \| 1.93 (0.36; 10.24) \| 2.36 (1.29; 4.33) \| 2.24 (1.71; 2.92) \| 6.58 (1.99; 21.77) \| \| **BMI≥25 kg/m^2^, without disease^1^ of interest** \| 1.14 (1.08; 1.20) \| \| 1.14 (1.08; 1.20) \| \| 1.14 (1.08; 1.20) \| 1.24 (1.18; 1.30) \| 1.24 (1.18; 1.30) \| 1.24 (1.18; 1.30) \| \| **BMI≥25 kg/m^2^, with disease (joint effect)** \| 2.21 (1.53; 3.19) \| \| 1.92 (1.21; 3.04) \| \| 1.54 (0.55; 4.28) \| 2.85 (2.00; 4.04) \| 2.93 (2.35; 3.65) \| 6.52 (3.76; 11.30) \| \| **RERI (95%CI)** \| **0.19 (-0.63; 1.00)** \| \| **0.06 (-0.63; 0.76)** \| \| **-0.53 (-3.75; 2.68)** \| **0.24 (-1.09; 1.58)** \| **0.45 (-0.12; 1.03)** \| **-0.31 (-8.20; 7.59)** \| \| **BMI<30 kg/m^2^, without disease^1^ of interest** \| 1.00 (ref) \| \| 1.00 (ref) \| \| 1.00 (ref) \| 1.00 (ref) \| 1.00 (ref) \| 1.00 (ref) \| \| **BMI<30 kg/m^2^, with disease** \| 1.71 (1.23; 2.38) \| \| 1.57 (1.07; 2.32) \| \| 2.87 (1.35; 6.08) \| 2.23 (1.50; 3.34) \| 2.16 (1.73; 2.70) \| 4.47 (2.02; 9.84) \| \| **BMI≥30 kg/m^2^, without disease^1^ of interest** \| 1.13 (1.06; 1.21) \| \| 1.13 (1.06; 1.21) \| \| 1.14 (1.07; 1.22) \| 1.27 (1.21; 1.34) \| 1.26 (1.19; 1.32) \| 1.27 (1.21; 1.34) \| \| **BMI≥30 kg/m^2^, with disease (joint effect)** \| 2.05 (1.46; 2.87) \| \| 2.51 (1.55; 4.05) \| \| 2.54 (1.13; 5.73) \| 2.85 (1.97; 4.11) \| 3.36 (2.62; 4.31) \| 7.02 (3.91; 12.63) \| \| **RERI (95%CI)** \| **0.21 (-0.29; 0.70)** \| \| **0.80 (-0.17; 1.78)** \| \| **-0.46 (-2.72; 1.80)** \| **0.34 (-0.49; 1.18)** \| **0.94 (0.27; 1.61)** \| **2.29 (-2.22; 6.79)** \| |
| Notes: The model included BMI as a binary variable with an interaction term with the binary variable of the existence of the disease of interest and was adjusted by duration of comorbidities, education levels, smoking status, alcohol consumption, height, use of HRT, physical activity, healthy diet score, two binary variables of the two cardiometabolic conditions status not studied and stratified by centre, menopausal status and age at recruitment (5-year categories)  RERI_RR_ = RR_11_ − RR_10_ − RR_01_ + 1, where _11_ denotes being exposed to both factors (eg, overweight/obesity and T2D), _10_ to one factor (eg, overweight/obesity), and _01_ to the other one (eg, *T2D*). A RERI of 0 was considered a lack of additive interaction and 95%CIs were calculated as proposed by *Hosmer and Lemeshow*.  Abbreviations: BMI: Body mass index; T2D: Type 2 Diabetes; CVD: Cardiovascular diseases; RERI: Relative excessive Risk due to Interaction  ^1^ without diseases means without the combination of the disease(s) studied. |

| **Figure S9: Forest plot of the relative excess risk of all-cancers due to interaction (with 95% Cis) between overweight and/or obesity and incident cardiometabolic diseases in EPIC and UKB cohort and the results of the meta-analysis (random effect models), in women** |
| --- |
| 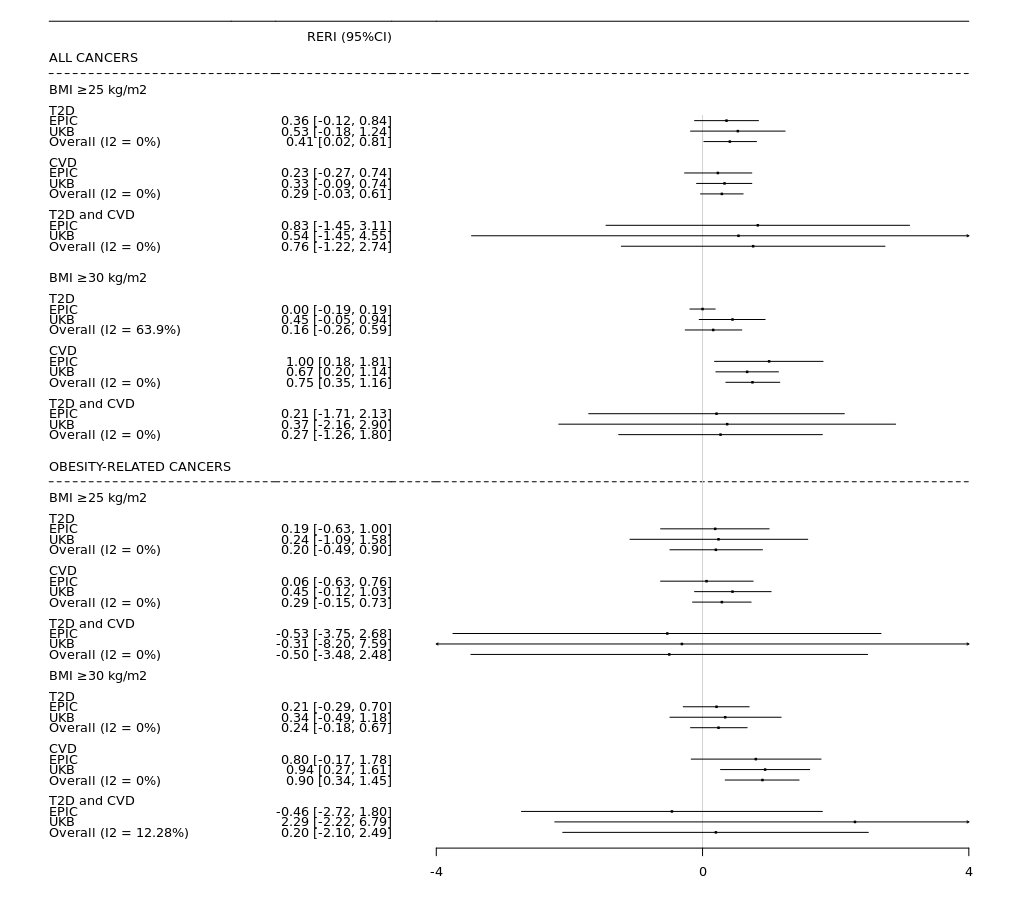 |
| I2 = I² index which quantify the dispersion of effect sizes in a meta-analysis  Abbreviations: SD : Standard deviation ; BMI: Body mass index; RERI: Relative excess risk due to interaction; 95%CI: 95% Confidence intervals; T2D : Type 2 Diabetes ; CVD: Cardiovascular diseases |

| **Table S11: Relative excess risk of all-cancers due to interaction between overweight and/or obesity and incident cardiometabolic diseases, in EPIC and UKB cohorts, among never smokers.** |
| --- |
| \|  \| **EPIC** \| \| \| \| \| **UKB** \| \| \| \| --- \| --- \| --- \| --- \| --- \| --- \| --- \| --- \| --- \| \| **T2D** \| \| **CVD** \| \| **T2D and CVD** \| **T2D** \| **CVD** \| **T2D and CVD** \| \| **ALL CANCERS** \| \| \| \| \| \| \| \| \| \| **BMI<25 kg/m^2^, without disease^1^ of interest** \| 1.00 (ref) \| 1.00 (ref) \| \| 1.00 (ref) \| \| 1.00 (ref) \| 1.00 (ref) \| 1.00 (ref) \| \| **BMI<25 kg/m^2^, with disease** \| 2.20 (1.39; 3.50) \| 1.82 (1.18; 2.81) \| \| 3.22 (1.02; 10.14) \| \| 1.62 (1.03; 2.53) \| 2.15 (2.05; 2.57) \| 1.69 (1.58; 6.14) \| \| **BMI≥25 kg/m^2^, without disease^1^ of interest** \| 1.05 (1.01; 1.11) \| 1.05 (1.00; 1.11) \| \| 1.04 (1.00; 1.11) \| \| 1.11 (1.08; 1.15) \| 1.11 (1.05; 1.11) \| 1.11 (1.06; 1.11) \| \| **BMI≥25 kg/m^2^, with disease (joint effect)** \| 1.89 (1.37; 2.61) \| 1.82 (1.25; 2.64) \| \| 2.02 (0.93; 4.39) \| \| 1.88 (1.43; 2.48) \| 2.62 (2.27; 3.01) \| 4.78 (3.21; 5.28) \| \| **RERI (95%CI)** \| **-0.37 (-1.22; 0.48)** \| **-0.06 (-0.71; 0.58)** \| \| **-1.26 (-4.84; 2.31)** \| \| **0.12 (-0.49; 0.80)** \| **0.35 (0.01; 0.70)** \| **2.98 (0.14; 5.82)** \| \| **BMI<30 kg/m^2^, without disease^1^ of interest** \| 1.00 (ref) \| \| 1.00 (ref) \| \| 1.00 (ref) \| 1.00 (ref) \| 1.00 (ref) \| 1.00 (ref) \| \| **BMI<30 kg/m^2^, with disease** \| 1.92 (1.37; 2.69) \| \| 1.74 (1.21; 2.52) \| \| 1.87 (0.75; 4.73) \| 1.67 (1.23; 2.26) \| 2.22 (1.93; 2.56) \| 4.57 (2.81; 7.42) \| \| **BMI≥30 kg/m^2^, without disease^1^ of interest** \| 1.13 (1.06; 1.20) \| \| 1.12 (1.05; 1.19) \| \| 1.12 (1.05; 1.19) \| 1.11 (1.06; 1.15) \| 1.10 (1.05; 1.14) \| 1.11 (1.07; 1.15) \| \| **BMI≥30 kg/m^2^, with disease (joint effect)** \| 1.90 (1.35; 2.67) \| \| 2.00 (1.26; 3.20) \| \| 2.57 (1.10; 6.04) \| 1.85 (1.38; 2.48) \| 2.78 (2.35; 3.28) \| 4.00 (2.51; 6.38) \| \| **RERI (95%CI)** \| **-0.15 (-0.63; 0.33)** \| \| **0.14 (-0.61; 0.90)** \| \| **0.58 (-1.74; 2.91)** \| **0.07 (-0.35; 0.50)** \| **0.45 (0.07; 0.83)** \| **-0.67 (-2.95; 1.61)** \| \| **OBESITY-RELATED CANCERS** \| \| \| \| \| \| \| \| \| \| **BMI<25 kg/m^2^, without disease^1^ of interest** \| 1.00 (ref) \| \| 1.00 (ref) \| \| 1.00 (ref) \| 1.00 (ref) \| 1.00 (ref) \| 1.00 (ref) \| \| **BMI<25 kg/m^2^, with disease** \| 1.66 (0.80; 3.42) \| \| 1.82 (0.95; 3.52) \| \| 1.89 (0.21; 16.93) \| 2.45 (1.30; 4.60) \| 2.99 (2.30; 3.88) \| 5.91 (1.38; 25.32) \| \| **BMI≥25 kg/m^2^, without disease^1^ of interest** \| 1.14 (1.06; 1.23) \| \| 1.15 (1.07; 1.23) \| \| 1.15 (1.07; 1.23) \| 1.24 (1.17; 1.30) \| 1.23 (1.18; 1.31) \| 1.24 (1.17; 1.30) \| \| **BMI≥25 kg/m^2^, with disease (joint effect)** \| 2.26 (1.44; 3.55) \| \| 1.92 (1.06; 3.47) \| \| 1.49 (0.38; 5.76) \| 2.61 (1.77; 3.85) \| 3.43 (2.97; 4.23) \| 8.07 (4.57; 14.26) \| \| **RERI (95%CI)** \| **0.46 (-0.66; 1.58)** \| \| **-0.05 (-0.99; 0.89)** \| \| **-0.55 (-4.82; 3.72)** \| **-0.08 (-1.45; 1.30)** \| **0.18 (-0.53; 0.90)** \| **1.93 (-6.94; 10.80)** \| \| **BMI<30 kg/m^2^, without disease^1^ of interest** \| 1.00 (ref) \| \| 1.00 (ref) \| \| 1.00 (ref) \| 1.00 (ref) \| 1.00 (ref) \| 1.00 (ref) \| \| **BMI<30 kg/m^2^, with disease** \| 1.99 (1.23; 3.22) \| \| 1.73 (0.97; 3.09) \| \| 0.76 (0.09; 6.35) \| 2.22 (1.44; 3.43) \| 2.73 (2.21; 3.38) \| 7.38 (3.65; 14.94) \| \| **BMI≥30 kg/m^2^, without disease^1^ of interest** \| 1.20 (1.10; 1.31) \| \| 1.20 (1.10; 1.30) \| \| 1.19 (1.10; 1.30) \| 1.33 (1.26; 1.41) \| 1.32 (1.24; 1.40) \| 1.33 (1.25; 1.41) \| \| **BMI≥30 kg/m^2^, with disease (joint effect)** \| 2.19 (1.36; 3.53) \| \| 2.02 (0.99; 4.13) \| \| 2.20 (0.56; 8.63) \| 2.57 (1.70; 3.88) \| 3.89 (3.03; 5.00) \| 7.24 (3.80; 13.79) \| \| **RERI (95%CI)** \| **-0.003 (-0.77; 0.76)** \| \| **0.09 (-1.03; 1.21)** \| \| **1.24 (-1.90; 4.39)** \| **0.02(-0.82; 0.86)** \| **0.83 (0.03; 1.64)** \| **-0.48 (-5.99; 5.04)** \| |
| Notes: The model included BMI as a binary variable with an interaction term with the binary variable of the existence of the disease of interest and was adjusted by duration of comorbidities, education levels, alcohol consumption, height, use of HRT, menopausal status, physical activity, healthy diet score, two binary variables of the two cardiometabolic conditions status not studied and stratified by centre, sex and age at recruitment (5-year categories)  RERI_RR_ = RR_11_ − RR_10_ − RR_01_ + 1, where _11_ denotes being exposed to both factors (eg, overweight/obesity and T2D), _10_ to one factor (eg, overweight/obesity), and _01_ to the other one (eg, *T2D*). A RERI of 0 was considered a lack of additive interaction and 95%CIs were calculated as proposed by *Hosmer and Lemeshow*.  Abbreviations: BMI: Body mass index; T2D: Type 2 Diabetes; CVD: Cardiovascular diseases; RERI: Relative excessive Risk due to Interaction  ^1^ without diseases means without the combination of the disease(s) studied. |

| **Figure S10: Forest plot of the relative excess risk of all-cancers due to interaction (with 95% CIs) between overweight and/or obesity and incident cardiometabolic diseases, in EPIC and UKB cohorts and the results of the meta-analysis (random effect models)** |
| --- |
| 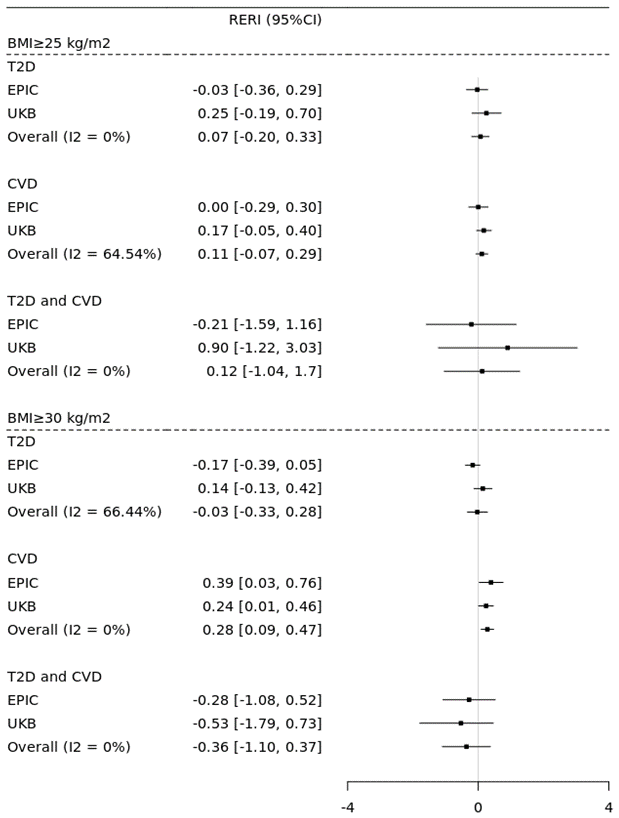 |
| I2 = I² index which quantify the dispersion of effect sizes in a meta-analysis  Abbreviations: RERI: Relative excess risk due to interaction; 95%CI: 95% Confidence intervals; BMI: Body mass index; T2D : Type 2 Diabetes ; CVD: Cardiovascular diseases |

| **Table S12: Relative excess risk of all-cancers due to interaction between overweight and/or obesity and incident cardiometabolic diseases, in EPIC and UKB cohorts** |
| --- |
| \|  \| **EPIC** \| \| \| \| \| **UKB** \| \| \| \| --- \| --- \| --- \| --- \| --- \| --- \| --- \| --- \| --- \| \| **T2D** \| \| **CVD** \| \| **T2D and CVD** \| **T2D** \| **CVD** \| **T2D and CVD** \| \| **BMI<25 kg/m^2^, without disease^1^ of interest** \| 1.00 (ref) \| 1.00 (ref) \| \| 1.00 (ref) \| \| 1.00 (ref) \| 1.00 (ref) \| 1.00 (ref) \| \| **BMI<25 kg/m^2^, with disease** \| 1.57 (1.21; 2.03) \| 1.67 (1.34; 2.08) \| \| 2.23 (1.17; 4.24) \| \| 1.60 (1.18; 2.16) \| 2.29 (2.04; 2.56) \| 3.06 (1.55; 6.03) \| \| **BMI≥25 kg/m^2^, without disease^1^ of interest** \| 1.00 (0.97; 1.04) \| 1.00 (0.97; 1.04) \| \| 1.00 (0.97; 1.04) \| \| 1.08 (1.05; 1.10) \| 1.08 (1.05; 1.10) \| 1.08 (1.05; 1.10) \| \| **BMI≥25 kg/m^2^, with disease (joint effect)** \| 1.54 (1.27; 1.86) \| 1.68 (1.38; 2.04) \| \| 2.02 (1.42; 2.87) \| \| 1.93 (1.62; 2.29) \| 2.54 (2.32;2.78) \| 4.05 (3.16; 5.19) \| \| **RERI (95%CI)** \| **-0.03 (-0.36; 0.29)** \| **0.00 (-0.29; 0.30)** \| \| **-0.21 (-1.59; 1.16)** \| \| **0.25 (-0.19; 0.70)** \| **0.17 (-0.05; 0.40)** \| **0.90 (-1.22; 3.03)** \| \|  \| \| \| \| \| \| \| \| \| \| **BMI<30 kg/m^2^, without disease^1^ of interest** \| 1.00 (ref) \| \| 1.00 (ref) \| \| 1.00 (ref) \| 1.00 (ref) \| 1.00 (ref) \| 1.00 (ref) \| \| **BMI<30 kg/m^2^, with disease** \| 1.87 (1.61; 2.18) \| \| 1.55 (1.32; 1.84) \| \| 2.66 (1.99; 3.56) \| 1.72 (1.42; 2.07) \| 2.30 (2.10; 2.51) \| 4.07 (3.01; 5.50) \| \| **BMI≥30 kg/m^2^, without disease^1^ of interest** \| 1.08 (1.04; 1.12) \| \| 1.06 (1.02; 1.10) \| \| 1.07 (1.03; 1.11) \| 1.05 (1.02; 1.08) \| 1.05 (1.02; 1.08) \| 1.06 (1.03; 1.09) \| \| **BMI≥30 kg/m^2^, with disease (joint effect)** \| 1.79 (1.52; 2.10) \| \| 2.02 (1.62; 2.51) \| \| 2.46 (1.76; 3.44) \| 1.91 (1.59; 2.30) \| 2.59 (2.32; 2.88) \| 3.60 (2.70; 4.79) \| \| **RERI (95%CI)** \| **-0.17 (-0.39; 0.05)** \| \| **0.39 (0.03; 0.76)** \| \| **-0.28 (-1.08; 0.52)** \| **0.14 (-0.13; 0.42)** \| **0.24 (0.01; 0.46)** \| **-0.53 (-1.79; 0.73)** \| |
| Notes: The model included BMI as a binary variable with an interaction term with the binary variable of the existence of the disease of interest and was adjusted by duration of comorbidities, education levels, smoking status, alcohol consumption, height, use of HRT, menopausal status, physical activity, healthy diet score, two binary variables of the two cardiometabolic conditions status not studied and stratified by sex, centre and age at recruitment (5-year categories)  RERI_RR_ = RR_11_ − RR_10_ − RR_01_ + 1, where _11_ denotes being exposed to both factors (eg, overweight/obesity and T2D), _10_ to one factor (eg, overweight/obesity), and _01_ to the other one (eg, *T2D*). A RERI of 0 was considered a lack of additive interaction and 95%CIs were calculated as proposed by *Hosmer and Lemeshow*.  Abbreviations: BMI: Body mass index; T2D: Type 2 Diabetes; CVD: Cardiovascular diseases; RERI: Relative excessive Risk due to Interaction  ^1^ without diseases means without the combination of the disease(s) studied. |

| **Table S13: Relative excess risk of all-cancers due to interaction between overweight and/or obesity and incident cardiometabolic diseases, in EPIC and UKB cohorts. Models further adjusted on metformin use** |
| --- |
| \|  \| **UKB** \| \| \| \| --- \| --- \| --- \| --- \| \| **T2D** \| **CVD** \| **T2D and CVD** \| \| **ALL CANCERS** \| \| \| \| \| **BMI<25 kg/m^2^, without disease^1^ of interest** \| 1.00 (ref) \| 1.00 (ref) \| 1.00 (ref) \| \| **BMI<25 kg/m^2^, with disease** \| 1.70 (1.26; 2.30) \| 2.29 (2.04; 2.56) \| 3.21 (1.63; 6.33) \| \| **BMI≥25 kg/m^2^, without disease^1^ of interest** \| 1.08 (1.05; 1.11) \| 1.08 (1.05; 1.11) \| 1.08 (1.06; 1.11) \| \| **BMI≥25 kg/m^2^, with disease (joint effect)** \| 2.09 (1.76; 2.49) \| 2.54 (2.32; 2.78) \| 4.30 (3.36; 5.52) \| \| **RERI (95%CI)** \| **0.31 (-0.16; 0.78)** \| **0.17 (-0.06; 0.40)** \| **1.01 (-1.23; 3.24)** \| \| **BMI<30 kg/m^2^, without disease^1^ of interest** \| 1.00 (ref) \| 1.00 (ref) \| 1.00 (ref) \| \| **BMI<30 kg/m^2^, with disease** \| 1.85 (1.53; 2.24) \| 2.29 (2.10; 2.51) \| 4.29 (3.17; 5.79) \| \| **BMI≥30 kg/m^2^, without disease^1^ of interest** \| 1.06 (1.03; 1.09) \| 1.06 (1.03; 1.09) \| 1.06 (1.04; 1.09) \| \| **BMI≥30 kg/m^2^, with disease (joint effect)** \| 2.08 (1.73; 2.50) \| 2.59 (2.32; 2.88) \| 3.84 (2.88; 5.12) \| \| **RERI (95%CI)** \| **0.16 (-0.13; 0.46)** \| **0.23 (0.005; 0.46)** \| **-0.51 (-1.85; 0.83)** \| \| **OBESITY-RELATED CANCERS** \| \| \| \| \| **BMI<25 kg/m^2^, without disease^1^ of interest** \| 1.00 (ref) \| 1.00 (ref) \| 1.00 (ref) \| \| **BMI<25 kg/m^2^, with disease** \| 2.35 (1.49; 3.72) \| 2.68 (2.23; 3.23) \| 6.64 (2.65; 16.61) \| \| **BMI≥25 kg/m^2^, without disease^1^ of interest** \| 1.23 (1.18; 1.28) \| 1.23 (1.18; 1.28) \| 1.23 (1.18; 1.28) \| \| **BMI≥25 kg/m^2^, with disease (joint effect)** \| 3.03 (2.34; 3.91) \| 3.42 (2.95; 3.95) \| 8.19 (5.67; 11.84) \| \| **RERI (95%CI)** \| **0.44 (-0.58; 1.46)** \| **0.50 (0.02; 0.98)** \| **1.33 (-4.87; 7.53)** \| \| **BMI<30 kg/m^2^, without disease^1^ of interest** \| 1.00 (ref) \| 1.00 (ref) \| 1.00 (ref) \| \| **BMI<30 kg/m^2^, with disease** \| 2.55 (1.92; 3.38) \| 2.68 (2.32; 3.10) \| 7.38 (4.67; 11.64) \| \| **BMI≥30 kg/m^2^, without disease^1^ of interest** \| 1.25 (1.20; 1.30) \| 1.24 (1.19; 1.29) \| 1.25 (1.20; 1.30) \| \| **BMI≥30 kg/m^2^, with disease (joint effect)** \| 2.86 (2.18; 3.75) \| 3.59 (3.02; 4.25) \| 7.33 (4.84; 11.08) \| \| **RERI (95%CI)** \| **0.07 (-0.56; 0.70)** \| **0.66 (0.16; 1.16)** \| **-0.30 (-3.86; 3.27)** \| |
| Notes: The model included BMI as a binary variable with an interaction term with the binary variable of the existence of the disease of interest and was adjusted by duration of comorbidities, education levels, smoking status, alcohol consumption, height, use of HRT, menopausal status, physical activity, metformin, healthy diet score, two binary variables of the two cardiometabolic conditions status not studied and stratified by centre and age at recruitment (5-year categories)  RERI_RR_ = RR_11_ − RR_10_ − RR_01_ + 1, where _11_ denotes being exposed to both factors (eg, overweight/obesity and T2D), _10_ to one factor (eg, overweight/obesity), and _01_ to the other one (eg, *T2D*). A RERI of 0 was considered a lack of additive interaction and 95%CIs were calculated as proposed by *Hosmer and Lemeshow*.  Abbreviations: BMI: Body mass index; T2D: Type 2 Diabetes; CVD: Cardiovascular diseases; RERI: Relative excessive Risk due to Interaction  ^1^ without diseases means without the combination of the disease(s) studied. |

| **Table S14: Relative excess risk of all-cancers due to interaction between overweight and/or obesity and incident cardiometabolic diseases, in EPIC and UKB cohorts. Models further adjusted on statins use** |
| --- |
| \|  \| **UKB** \| \| \| \| --- \| --- \| --- \| --- \| \| **T2D** \| **CVD** \| **T2D and CVD** \| \| **ALL CANCERS** \| \| \| \| \| **BMI<25 kg/m^2^, without disease^1^ of interest** \| 1.00 (ref) \| 1.00 (ref) \| 1.00 (ref) \| \| **BMI<25 kg/m^2^, with disease** \| 1.63 (1.20; 2.19) \| 2.30 (2.05; 2.57) \| 3.12 (1.58; 6.14) \| \| **BMI≥25 kg/m^2^, without disease^1^ of interest** \| 1.08 (1.05; 1.11) \| 1.08 (1.05; 1.11) \| 1.08 (1.06; 1.11) \| \| **BMI≥25 kg/m^2^, with disease (joint effect)** \| 1.97 (1.66; 2.34) \| 2.56 (2.34; 2.80) \| 4.12 (3.21; 5.28) \| \| **RERI (95%CI)** \| **0.26 (-0.18; 0.71)** \| **0.18 (-0.05; 0.41)** \| **0.92 (-1.24; 3.08)** \| \| **BMI<30 kg/m^2^, without disease^1^ of interest** \| 1.00 (ref) \| 1.00 (ref) \| 1.00 (ref) \| \| **BMI<30 kg/m^2^, with disease** \| 1.75 (1.45; 2.11) \| 2.31 (2.11; 2.52) \| 4.13 (3.05; 5.58) \| \| **BMI≥30 kg/m^2^, without disease^1^ of interest** \| 1.06 (1.03; 1.09) \| 1.06 (1.03; 1.09) \| 1.06 (1.04; 1.09) \| \| **BMI≥30 kg/m^2^, with disease (joint effect)** \| 1.95 (1.63; 2.34) \| 2.61 (2.34; 2.90) \| 3.65 (2.74; 5.58) \| \| **RERI (95%CI)** \| **0.14 (-0.14; 0.42)** \| **0.24 (0.01; 0.47)** \| **-0.54 (-1.82; 0.74)** \| \| **OBESITY-RELATED CANCERS** \| \| \| \| \| **BMI<25 kg/m^2^, without disease^1^ of interest** \| 1.00 (ref) \| 1.00 (ref) \| 1.00 (ref) \| \| **BMI<25 kg/m^2^, with disease** \| 2.30 (1.45; 3.64) \| 2.69 (2.23; 3.24) \| 6.51 (2.60; 16.29) \| \| **BMI≥25 kg/m^2^, without disease^1^ of interest** \| 1.23 (1.18; 1.28) \| 1.23 (1.18; 1.28) \| 1.23 (1.18; 1.28) \| \| **BMI≥25 kg/m^2^, with disease (joint effect)** \| 2.93 (2.28; 3.78) \| 3.43 (2.97; 3.96) \| 8.00 (5.54; 11.54) \| \| **RERI (95%CI)** \| **0.40 (-0.59; 1.40)** \| **0.51 (0.03; 0.99)** \| **1.26 (-4.82; 7.33)** \| \| **BMI<30 kg/m^2^, without disease^1^ of interest** \| 1.00 (ref) \| 1.00 (ref) \| 1.00 (ref) \| \| **BMI<30 kg/m^2^, with disease** \| 2.46 (1.86; 3.27) \| 2.69 (2.33; 3.11) \| 7.19 (4.56; 11.35) \| \| **BMI≥30 kg/m^2^, without disease^1^ of interest** \| 1.25 (1.20; 1.31) \| 1.24 (1.19; 1.29) \| 1.25 (1.20; 1.30) \| \| **BMI≥30 kg/m^2^, with disease (joint effect)** \| 2.76 (2.11; 3.61) \| 3.60 (3.03; 4.26) \| 7.11 (4.70; 10.75) \| \| **RERI (95%CI)** \| **0.05(-0.56; 0.66)** \| **0.67 (0.17; 1.17)** \| **-0.33 (-3.80; 3.14)** \| |
| Notes: The model included BMI as a binary variable with an interaction term with the binary variable of the existence of the disease of interest and was adjusted by duration of comorbidities, education levels, smoking status, alcohol consumption, height, use of HRT, menopausal status, physical activity, metformin, healthy diet score, two binary variables of the two cardiometabolic conditions status not studied and stratified by centre and age at recruitment (5-year categories)  RERI_RR_ = RR_11_ − RR_10_ − RR_01_ + 1, where _11_ denotes being exposed to both factors (eg, overweight/obesity and T2D), _10_ to one factor (eg, overweight/obesity), and _01_ to the other one (eg, *T2D*). A RERI of 0 was considered a lack of additive interaction and 95%CIs were calculated as proposed by *Hosmer and Lemeshow*.  Abbreviations: BMI: Body mass index; T2D: Type 2 Diabetes; CVD: Cardiovascular diseases; RERI: Relative excessive Risk due to Interaction  ^1^ without diseases means without the combination of the disease(s) studied. |
